# Supplementary figures and images for: Interferon regulatory factor 4 mediates nonenzymatic IRE1 dependency in multiple myeloma cells
Source: PLoS Biol. 2025 Apr 11;23(4):e3003096. doi: 10.1371/journal.pbio.3003096 (PMC12052183; doi:10.1371/journal.pbio.3003096)

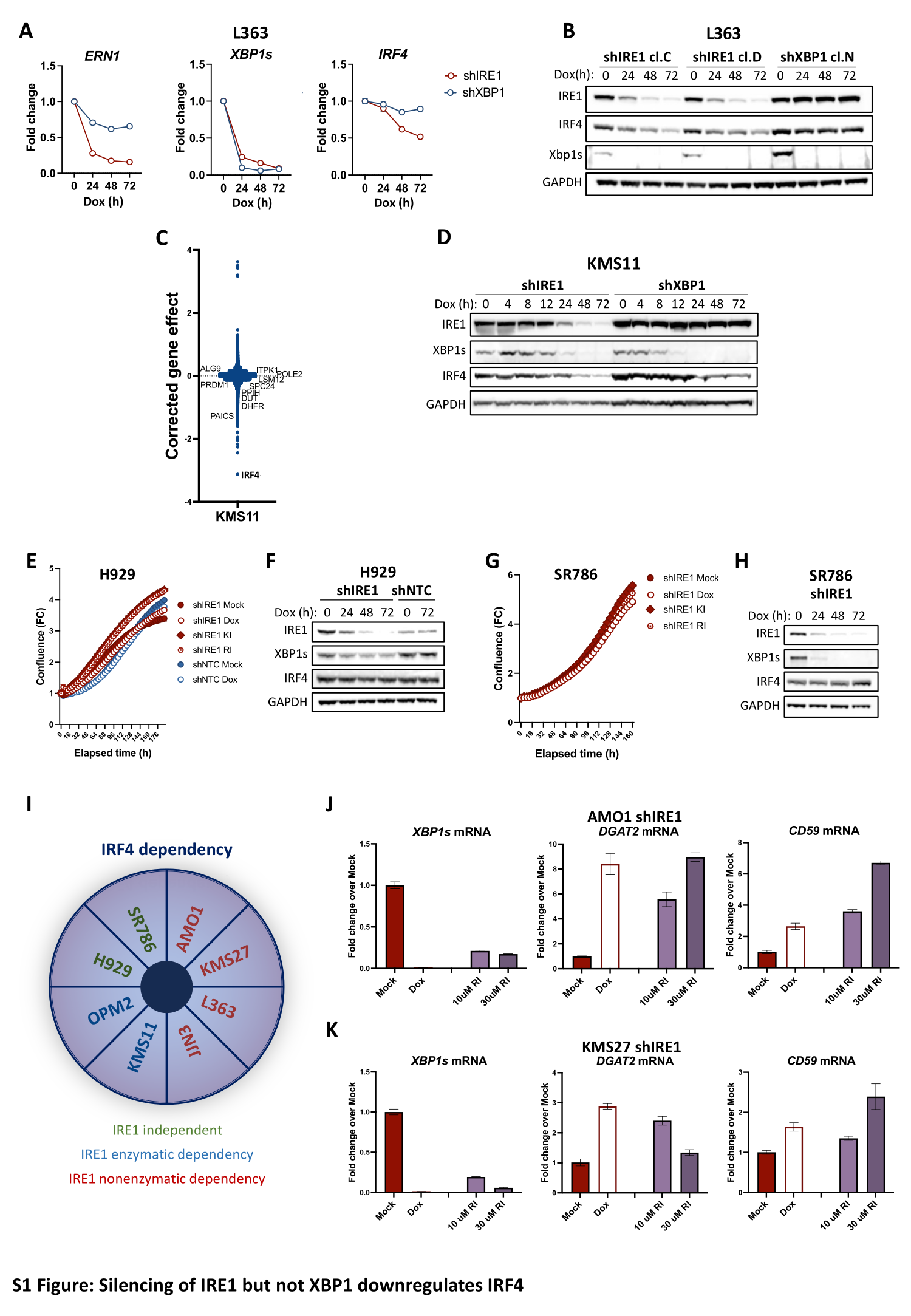

Supplement: S1 Fig — (A) IRF4 mRNA abundance in IRE1 or XBP1-depleted L363 cells. L363 shIRE1 Cl.C versus L363 shXBP1 Cl.N cells were treated with Dox (0.2 μg/mL) for the indicated time and analyzed by RT-qPCR for levels of ERN1, IRF4 and XBP1s. Data represented as mean ± SEM. (B) IRF4 protein abundance in IRE1 or XBP1-depleted L363 cells. L363 shIRE1 Cl.C or L363 shIRE1 Cl.D versus L363 shXBP1 Cl.N cells were treated with Dox (0.2 μg/mL) for the indicated time and were analyzed by IB for IRF4 protein levels. (C) Corrected gene effect scores of IRE1 codependencies, in KMS11: Chronos gene-effect values from KMS11 genes (derived from Depmap) were “corrected” to reveal MM-specific dependencies. Annotated are genes decreased 2-fold or more by IRE1 silencing in AMO1 cells (red in Fig 1A). (D) IRF4 protein abundance in IRE1 or XBP1-depleted KMS11 cells. KMS11 shIRE1 or KMS11 shXBP1 Cl.8 cells were treated with Dox (0.2 μg/mL) for the indicated time and analyzed by IB for IRF4 protein levels. (E) Effect of IRE1 silencing or inhibition on in vitro spheroid growth of H929 cells. Cells were stably transfected with plasmids encoding Dox-inducible shRNAs against either IRE1 (red) or non-targeting control (blue). Growth of these cells in the absence (closed symbols) or presence (open symbols) of Dox (0.2 μg/mL) was compared to that of cells expressing shNTC or cells treated with IRE1 RNase (RI; 1 μM) or kinase (KI; 1 μM) inhibitors. Spheroid growth, depicted as FC confluence, was monitored by time-lapse microscopy in an IncuCyte instrument and values represent mean ± SEM. (F) IRF4 protein abundance in IRE1-depleted H929 cells. H929 cells stably transfected with Dox-inducible shRNAs against IRE1 or non targeting control (NTC) were treated with Dox (0.2 μg/mL) for the indicated time and analyzed by IB for IRF4 protein levels. (G) Effect of IRE1 silencing on in vitro spheroid growth in IRE1-independent T-cell lymphoma, SR796 cells. Cells were stably transfected with plasmids encoding Dox-inducible shRN [file pbio.3003096.s001.tiff]

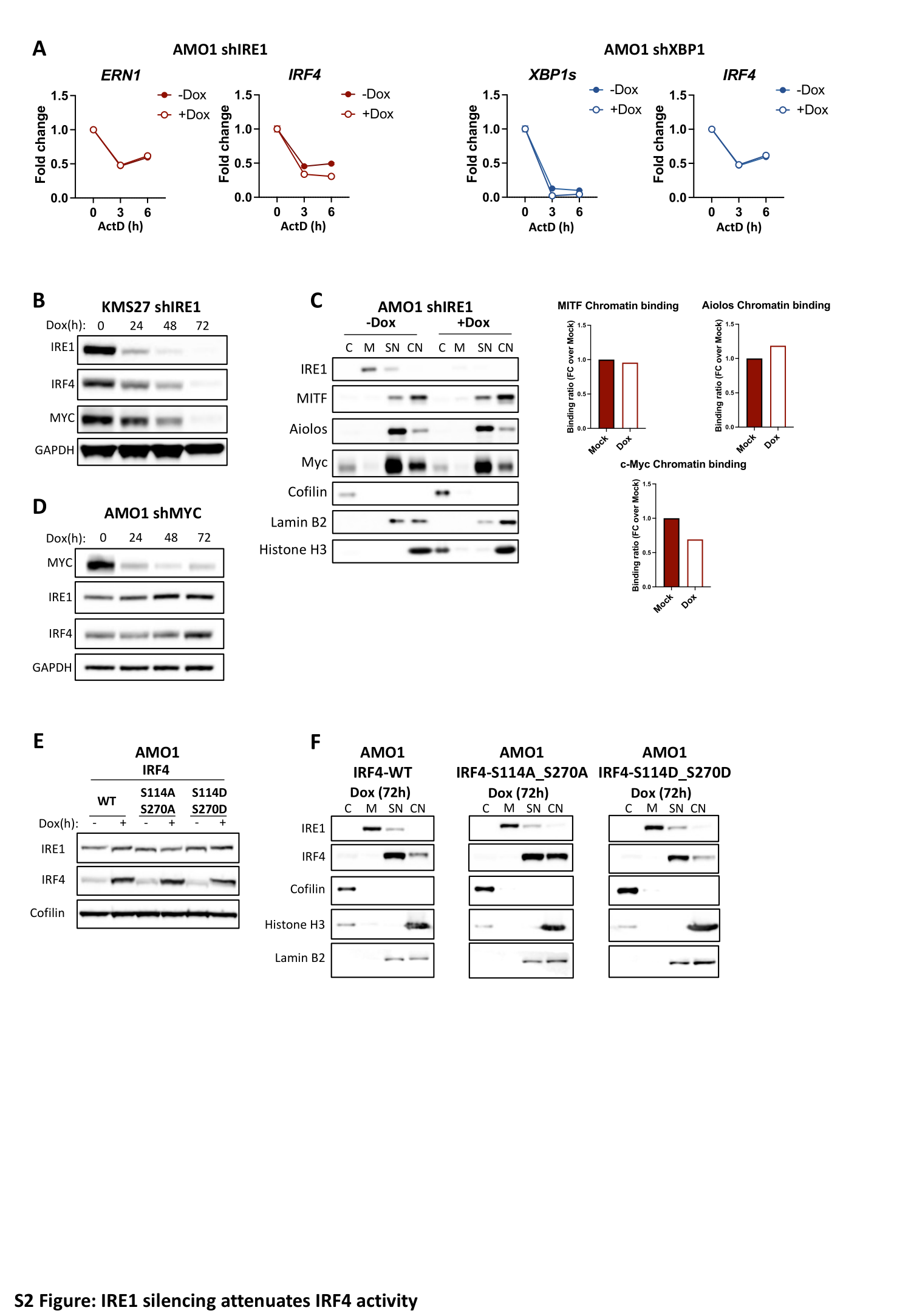

Supplement: S2 Fig — (A) Effect of IRE1 silencing on IRF4 mRNA stability. AMO1 shIRE1 Cl.1 (left) or shXBP1 Cl.1 (right) cells were pre-treated with or without 0.2 μg/mL Dox for 48 h and then 1 μg/mL Actinomycin D (ActD) was added for 3 h or 6 h. Samples were analyzed by RT-qPCR for ERN1, IRF4 and XBP1s. Data represented as mean ± SEM. (B) Effect of IRE1 silencing on Myc protein levels in KMS27. KMS27 shIRE1 cells underwent a Dox (0.2 μg/mL) time course and were analyzed by IB for IRF4 and Myc. (C) Effect of IRE1 silencing on IRF4-regulating transcription factor chromatin-binding activity. AMO1 shIRE1 Cl.1 cells were cultured in the absence or presence of 0.2 μg/mL Dox for 72 h. Cells were sequentially lysed into 4 subcellular fractions: C – cytoplasmic, M – Membrane, SN – Soluble Nuclear, CN – Chromatin-bound Nuclear. Nuclear fractions were analyzed by IB for IRE1, MITF, Myc, and Aiolos while Cofilin, Histone H3, and Lamin B2 serve as fractionation controls. Right: The ratio of chromatin-bound over soluble nuclear MITF, Aiolos, and Myc was determined by densitometry and is depicted relative to mock-treated cells. (D) Validation of MYC protein depletion by shMYC silencing. AMO1 samples from Fig 2E were analyzed by IB for MYC, IRE1, and IRF4. GAPDH used as a loading control. (E) Validation of WT and phospho-mutant IRF4 ectopic expression. AMO1 WT IRF4 versus AMO1 S114A_S270A versus S114D_270D were treated in the absence or presence of 0.2 μg/mL Dox for 72 h. The samples were analyzed by IB for IRF4 protein since the mutant IRF4 constructs are not recognized by complementary to endogenous IRF4 PCR primers. (F) Substitution of S114 and S270 by alanine versus aspartic acid and its effect on IRF4’s chromatin-binding activity. Samples from Fig 2H were analyzed by IB as in Fig 2F: C – cytoplasmic, M – Membrane, SN – Soluble Nuclear, CN – Chromatin-bound Nuclear and were analyzed by IB for IRF4 before this was quantified and normalized for Fig 2H. The data underlying this figure can be found in [file pbio.3003096.s002.tiff]

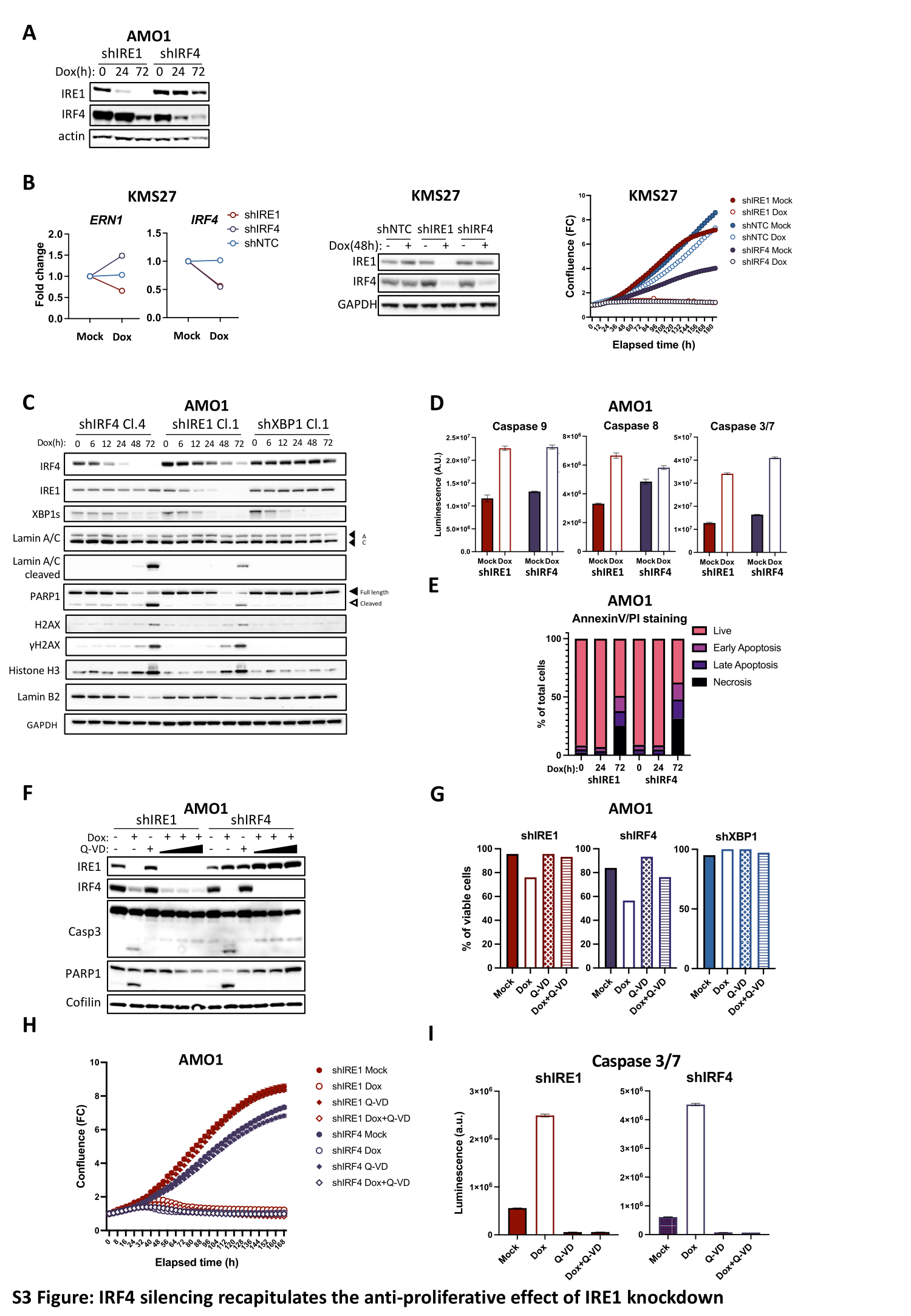

Supplement: S3 Fig — (A) Validation of IRF4 depletion by IRF4 silencing. AMO1 shIRE1 Cl.1 or shIRF4 Cl.1 cells were stably transfected with plasmids encoding Dox-inducible shRNAs against either IRF4 or IRE1. The cells were treated with Dox (0.2 μg/mL) for the indicated time. Samples were analyzed by IB for IRE1 and IRF4. (B) Effect of IRF4, IRE1 or NTC silencing on in vitro spheroid growth of KMS27 and validation of IRF4 silencing. KMS27 cells were stably transfected with plasmids encoding Dox-inducible shRNAs against either IRF4 (purple) or non-targeting control (blue). Left, middle: KMS27 shNTC, shIRE1, or shIRF4 cells were treated with Dox (0.2 μg/mL) for the indicated time. Samples were analyzed by RT-qPCR and IB for IRE1/ERN1 and IRF4. Right: Growth of these cells in the absence (closed symbols) or presence (open symbols) of Dox (0.2 μg/mL) was compared to that of cells expressing shRNAs against IRE1 or NTC. Spheroid growth, depicted as FC confluence, was monitored by time-lapse microscopy in an IncuCyte instrument and values represent mean ± SEM. (C) Cell death markers. AMO1 shIRE1 Cl.1, shIRF4 Cl.1, or shXBP1 Cl.1 cells were treated in the absence or presence of Dox (0.2 μg/mL) for up to 72 h and post-nuclear lysates were analyzed by IB for Lamin A/C and PARP1 cleavage as well as Histones. GAPDH is used as a loading control. (D) Effect of IRE1 or IRF4 silencing on caspase activation. AMO1 shIRE1 Cl.1 or shIRF4 Cl.1 cells treated in the absence (filled bars) or presence of Dox (0.2 μg/mL) for 72 h were analyzed for caspase activity by Caspase-Glo assays. Representative replicate. Values presented as mean ±SEM. (E) Effect of IRE1 or IRF4 silencing on Annexin V/ PI staining. AMO1 shIRE1 Cl.1 or shIRF4 Cl.1 cells were treated with Dox (0.2 μg/mL) for the indicated times. Cells were then stained with FITC-Annexin V and PI and analyzed by flow cytometry for early apoptotic (FITC+ PI-), late apoptotic (FITC+ PI+), and necrotic (FITC- PI+) cells. (F) Q-VD blockade of caspase cleavage dur [file pbio.3003096.s003.tiff]

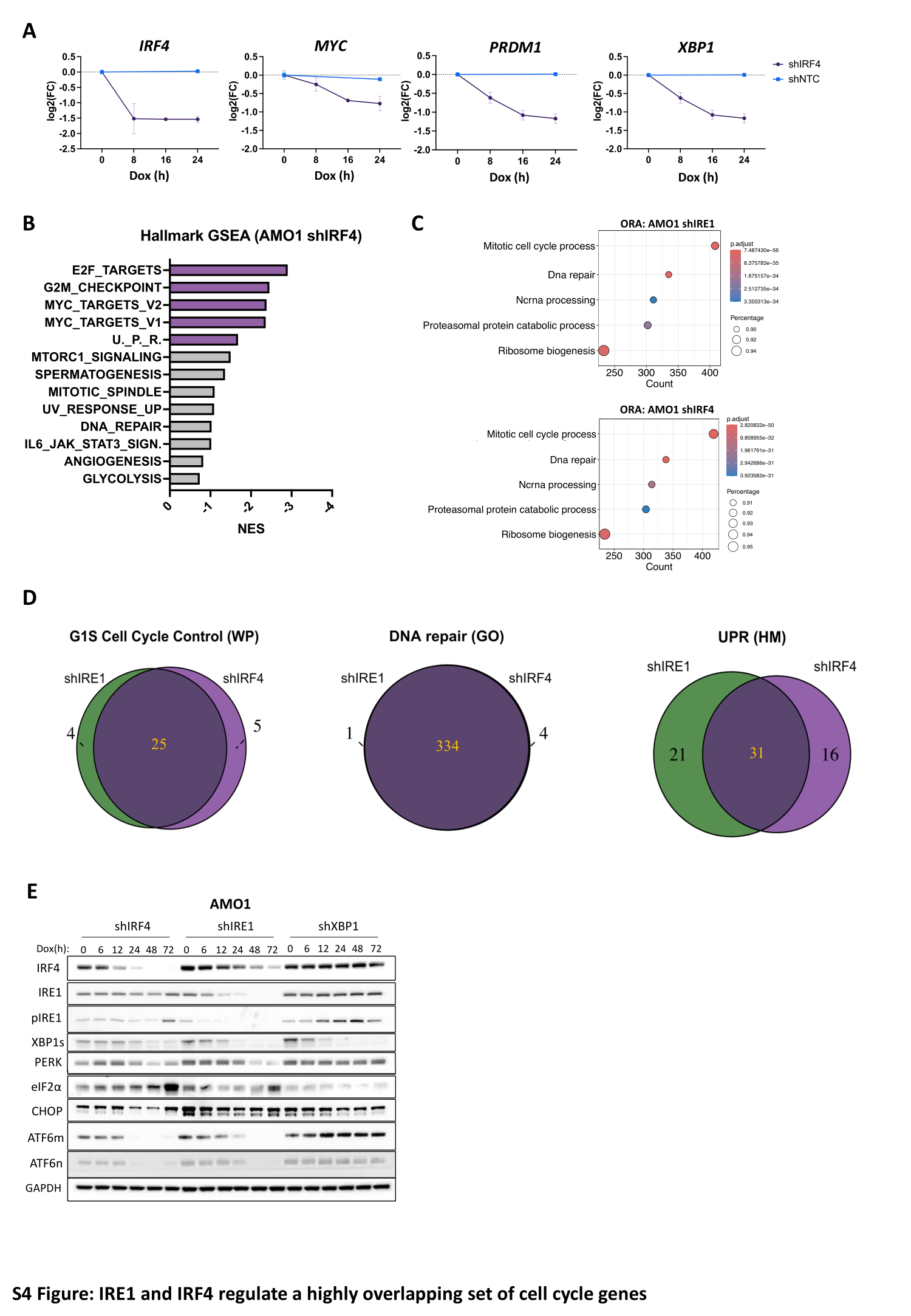

Supplement: S4 Fig — (A) Validation of samples from Fig 4. AMO1 shIRF4 Cl.1 or shNTC bulk RNA-seq analysis (addressed in Fig 4), validates that IRF4 transcripts were depleted during the time course of silencing, as well as transcripts of known IRF4 targets. i.e., MYC, PRDM1 (transcript of BLIMP1) and XBP1. (B) Complete IRF4 GSEA (Hallmark). GSEA analysis was performed as described in Fig 4A. Shown all enriched datasets. In gray, gene sets with FDR > 0.02. (C) ORA of AMO1 shIRE1 (top) and shIRF4 (bottom). Overrepresentation analysis of the transcriptomics results was performed using the Interactive analysis enrichment tool. Depicted are select GO terms. (D) Overlap of genes between IRE1 and IRF4 Knockdowns. After GSEA in Fig 4A, Leading Edge Genes (S2 Table), representing those contributing most significantly to the enrichment of the given gene sets, were extracted for both genetic backgrounds. Shown: Venn diagrams illustrating the intersection between the Leading-Edge Genes for “G1S Cell Cycle Control” (Wiki Pathways) and “Unfolded Protein Response” (UPR; Hallmark) between IRE1 knockdown (shIRE1) and IRF4 knockdown (shIRF4) backgrounds. Middle: Venn diagram representing all DNA repair GO term genes identified in the two backgrounds and their overlap. (E) UPR downregulation was validated by IB in both genetic backgrounds. Samples treated as in Fig 4D were analyzed by IB for pIRE1 as well as PERK and ATF6 pathway proteins. (F) Effect of IRE1 or IRF4 knockdown on mRNA expression of DNA repair genes. Heatmap depicting the top 100 downregulated genes match to “DNA repair” GO term in the transcriptomics analyses described in Fig 4A. (G) Complete IRF4 GSEA (Hallmark) analysis in KMS27 cells. Analysis was performed as described in Fig 4A for KMS27 cells. Shown all enriched datasets. In gray, gene sets with FDR > 0.02. Right: Overlap of Leading-Edge Genes from GSEA analyses in IRF4-deficient AMO1 and KMS27 cells. After GSEA in Fig 4A, Leading Edge Genes (S2 Table), representing those contributin [file pbio.3003096.s004.tiff]

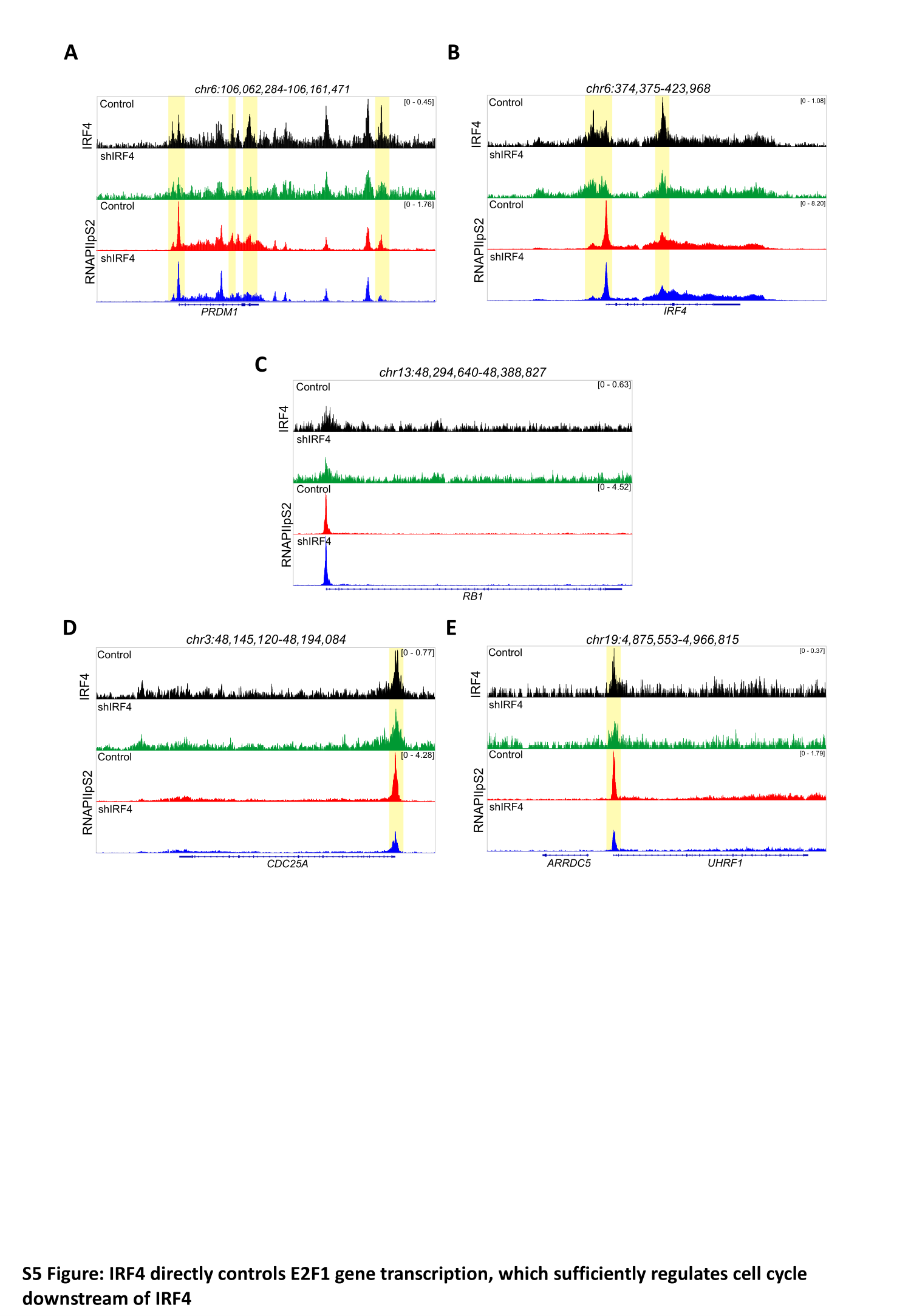

Supplement: S5 Fig — (A) IRF4 differential binding within the PRDM1 locus—a characterized IRF4 target—by ChIP-seq differential binding analysis. Genome browser tracks depicting the intensity of IRF4 (black and green) or RNAPIIpS2 (red and blue) binding in IRF4 proficient or deficient cells, respectively. Yellow shadowing indicates the differential binding regions identified by the analysis in Fig 5A. (B) IRF4 differential binding within the IRF4 locus by ChIP-seq differential binding analysis. Genome browser tracks depicting the intensity of IRF4 (black and green) or RNAPIIpS2 (red and blue) binding in IRF4 proficient or deficient cells, respectively. Yellow shadowing indicates the differential binding regions identified by the analysis in Fig 5A. (C) Genome browser depiction of ChIP-seq signals across RB1 locus. Genome browser tracks depicting the intensity of IRF4 (black and green) or RNAPIIpS2 (red and blue) binding in IRF4 proficient or deficient cells, respectively. No differential binding regions were identified by differential binding analysis. (D) IRF4 differential binding within the CDC25A locus by ChIP-seq differential binding analysis. Genome browser tracks depicting the intensity of IRF4 (black and green) or RNAPIIpS2 (red and blue) binding in IRF4 proficient or deficient cells, respectively. Yellow shadowing indicates the differential binding region identified by the analysis in Fig 5A. (E) IRF4 differential binding within the UHRF1 locus by ChIP-seq differential binding analysis. Genome browser tracks depicting the intensity of IRF4 (black and green) or RNAPIIpS2 (red and blue) binding in IRF4 proficient or deficient cells, respectively. Yellow shadowing indicates the differential binding region identified by the analysis in Fig 5A. The data underlying this figure can be found in GEO repository (GSE288671) and S2 Data. (TIFF) [file pbio.3003096.s005.tiff]

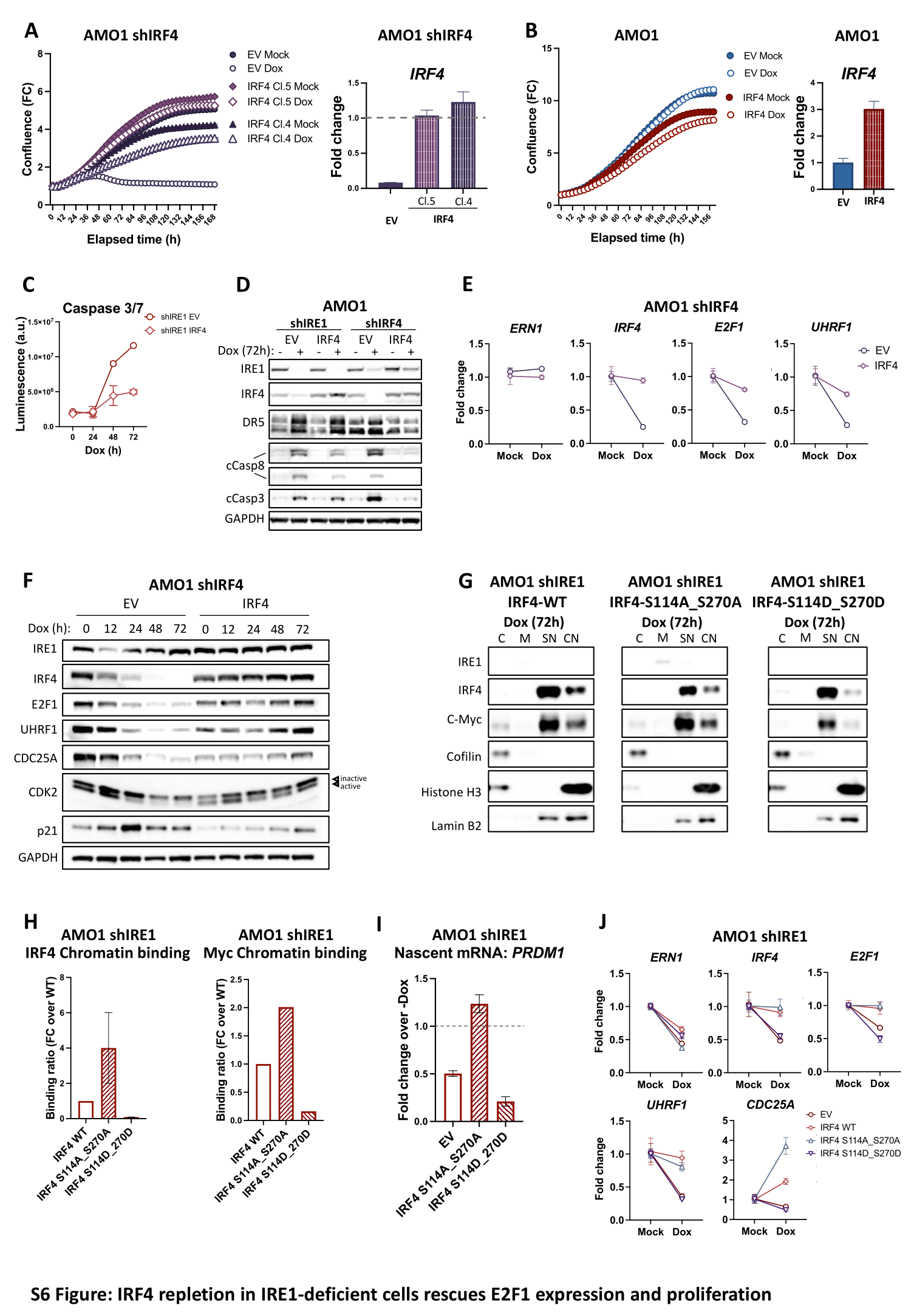

Supplement: S6 Fig — (A) Effect of IRF4 expression levels in the growth of IRF4 rescued cell lines. AMO1 shIRF4 cells were stably transfected with Dox-inducible IRF4 or EV. AMO1 shIRF4 EV or individual shIRF4 IRF4 clones were cultured in the absence (closed symbols) or presence (open symbols) of Dox (0.1 μg/mL). Left: Spheroid growth, depicted as FC confluence, was monitored by time-lapse microscopy in an IncuCyte instrument and values represent mean ± SEM. Right: Analysis of these cells after 24 h of culture in the same conditions by RT-qPCR for IRF4 mRNA levels. Dashed line represents the level of endogenous IRF4 expression. Values represent mean ± SEM. (B) Effect of ectopic IRF4 expression in the growth of AMO1 cells. AMO1 shNTC cells were stably transfected with inducible IRF4 or EV. The cells were then cultured in the absence (closed symbols) or presence (open symbols) of Dox (0.1 μg/mL) and spheroid growth, depicted as FC confluence, was monitored by time-lapse microscopy in an IncuCyte instrument and values represent mean ± SEM. Right: Analysis of these cells after 24 h of culture in the same conditions by RT-qPCR for IRF4 mRNA levels. Values represent mean ± SEM. (C) Caspase activation upon IRF4 re-expression in IRE1-deficient cells. AMO1 shIRE1 Cl.1 EV versus shIRE1 Cl.1 IRF4 Cl.4 cells were treated in the absence (filled symbols) or presence (open symbols) of Dox (0.2 μg/mL) for the indicated times were analyzed for caspase activity by Caspase 3/7-Glo assay. Values presented as mean ±SEM. (D) Effect of IRE1 silencing on DR5 levels compared to the effect of ectopic IRF4 expression in IRE1 deficient cells. AMO1 shIRE1 EV or IRF4 Cl.4 as well as AMO1 shIRF4 EV or IRF4 Cl.5 were incubated in the absence or presence of 0.1 μg/mL Dox for 72 h. The samples were then analyzed for DR5 levels by IB. Cleaved Caspases served as apoptosis markers. (E) Effect of IRF4 repletion on E2Fs and other targets during IRF4 silencing. AMO1 shIRF4 Cl.1 EV or shIRF4 Cl.1 IRF4 Cl. 5 cells were cultured [file pbio.3003096.s006.tiff]

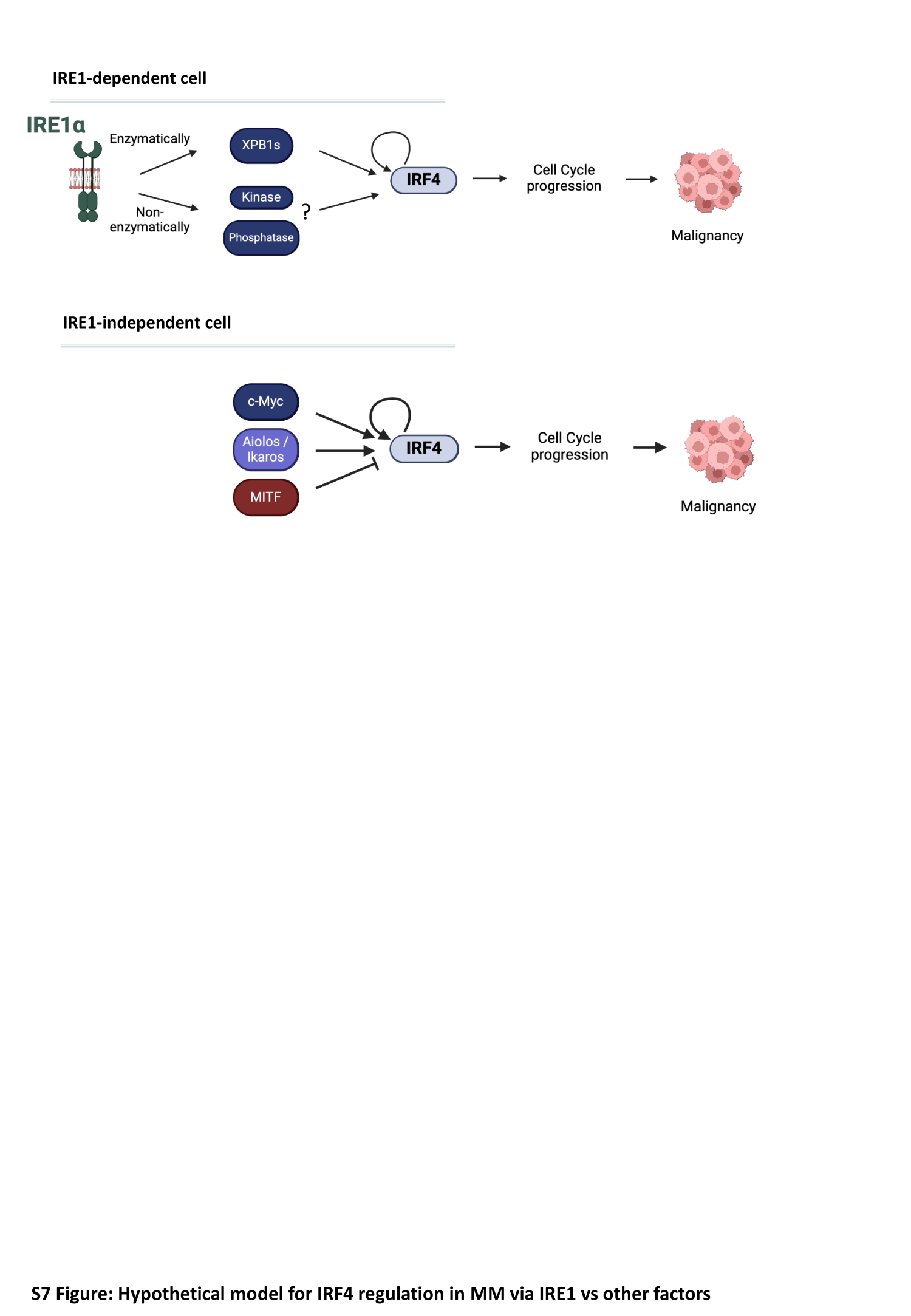

Supplement: S7 Fig — Schematic representation of IRE1 supporting IRF4 activity/expression in IRE1-dependent MM lines. Two newly identified modes of IRF4 regulation stemming from IRE1 require either XBP1s (enzymatic IRE1 dependency) or an unknown phosphotransferase intermediary that acts independently of IRE1 enzymatic activity (nonenzymatic dependency). IRE1-independent cell lines are dependent on IRF4 but IRF4 is not regulated by IRE1 but rather by previously identified transcription factors. The latter axes of IRF4 regulation may be present in IRE1-dependent cell lines as well. Created in BioRender. Oikonomidi, I. (2025) https://BioRender.com/b50x169. (TIFF) [file pbio.3003096.s007.tiff]
