# Supplementary material for: Interferon regulatory factor 4 mediates nonenzymatic IRE1 dependency in multiple myeloma cells
Source: PLoS Biol. 2025 Apr 11;23(4):e3003096. doi: 10.1371/journal.pbio.3003096 (PMC12052183; doi:10.1371/journal.pbio.3003096)
Supplement: S1 Raw images — Each blot is labeled to annotate sample order and antibody of blotting. Lanes not included in the final figures are marked with “X” above the lane label. (PDF) [file pbio.3003096.s008.pdf]

1F

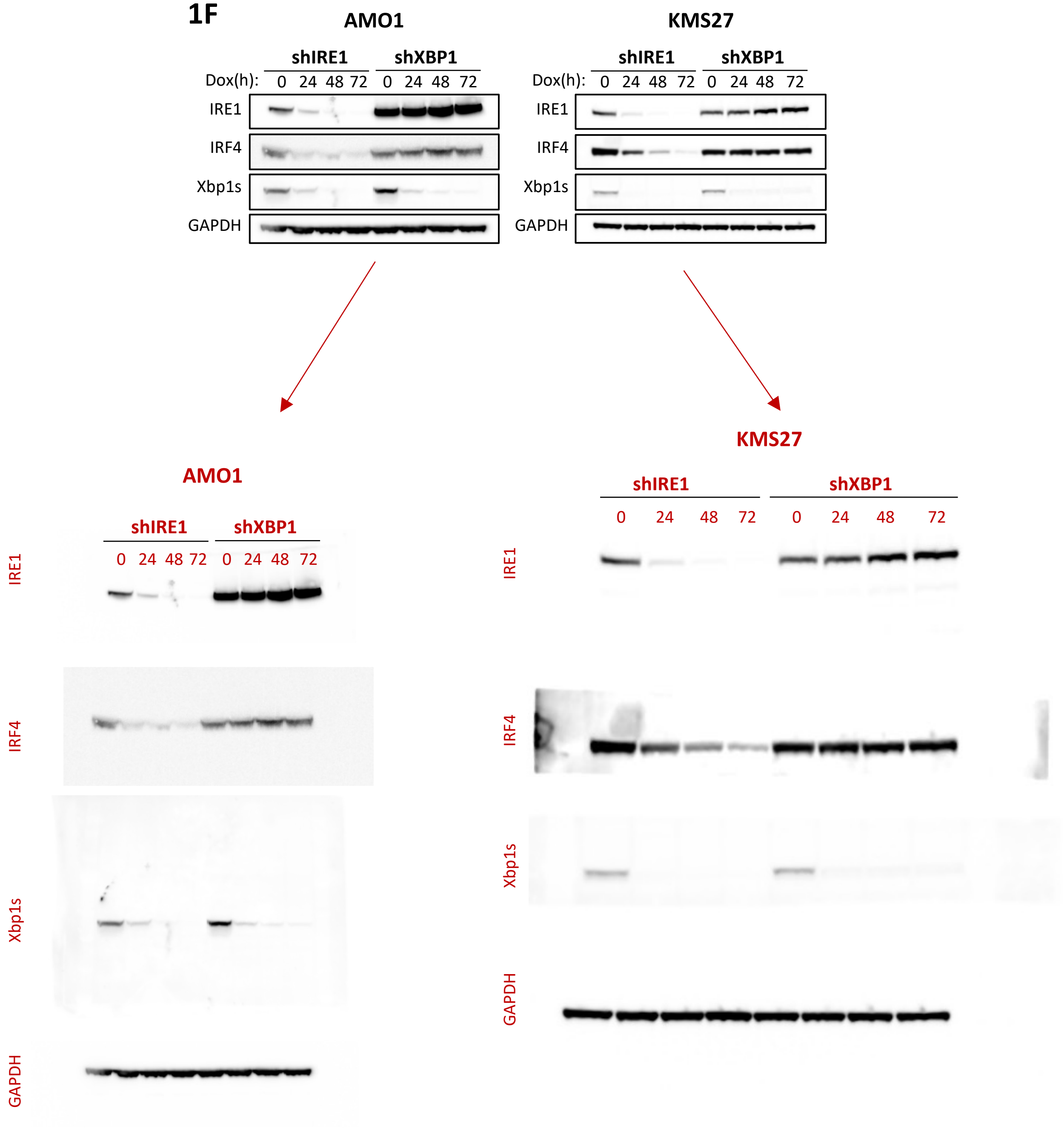

AMO1
KMS27
shIRE1
shXBP1
Dox(h):
0 24 48 72 0 24 48 72
IRE1
IRF4
Xbp1s
GAPDH
AMO1
shIRE1
shXBP1
0 24 48 72 0 24 48 72
IRE1
IRF4
Xbp1s
GAPDH
KMS27
shIRE1
shXBP1
0 24 48 72 0 24 48 72
IRE1
IRF4
Xbp1s
GAPDH

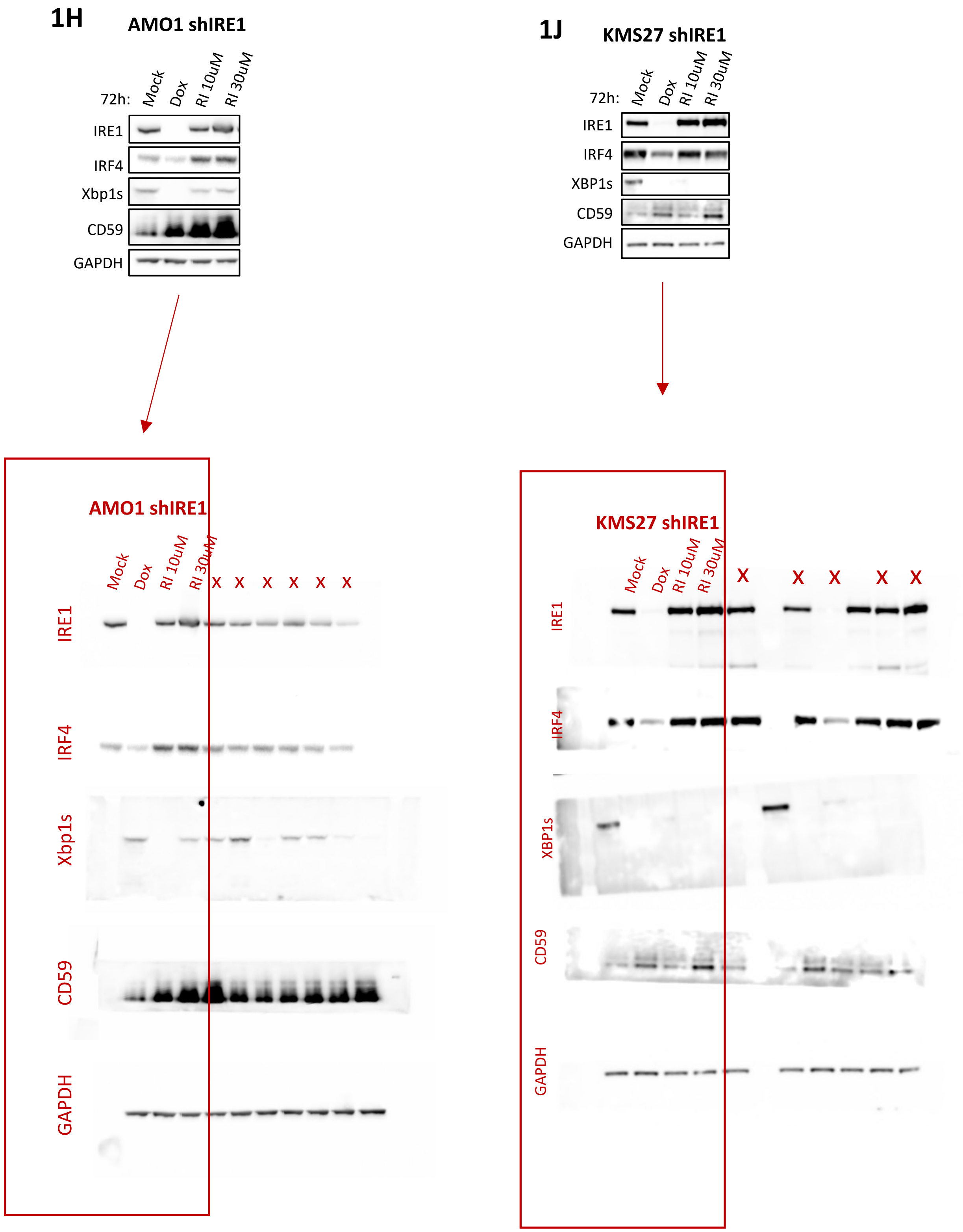
1H
AMO1 shIRE1
72h:
Mock
Dox
RI 10uM
RI 30uM
IRE1
IRF4
Xbp1s
CD59
GAPDH
1J
KMS27 shIRE1
72h:
Mock
Dox
RI 10uM
RI 30uM
IRE1
IRF4
XBP1s
CD59
GAPDH
AMO1 shIRE1
Mock
Dox
RI 10uM
RI 30uM
X X X X X X
IRE1
IRF4
Xbp1s
CD59
GAPDH
KMS27 shIRE1
Mock
Dox
RI 10uM
RI 30uM
X X X X X
IRE1
IRF4
XBP1s
CD59
GAPDH

S1B

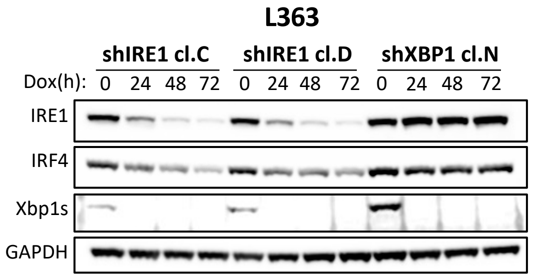
L363
shIRE1 cl.C
shIRE1 cl.D
shXBP1 cl.N
Dox(h): 0 24 48 72 0 24 48 72 0 24 48 72
IRE1
IRF4
Xbp1s
GAPDH

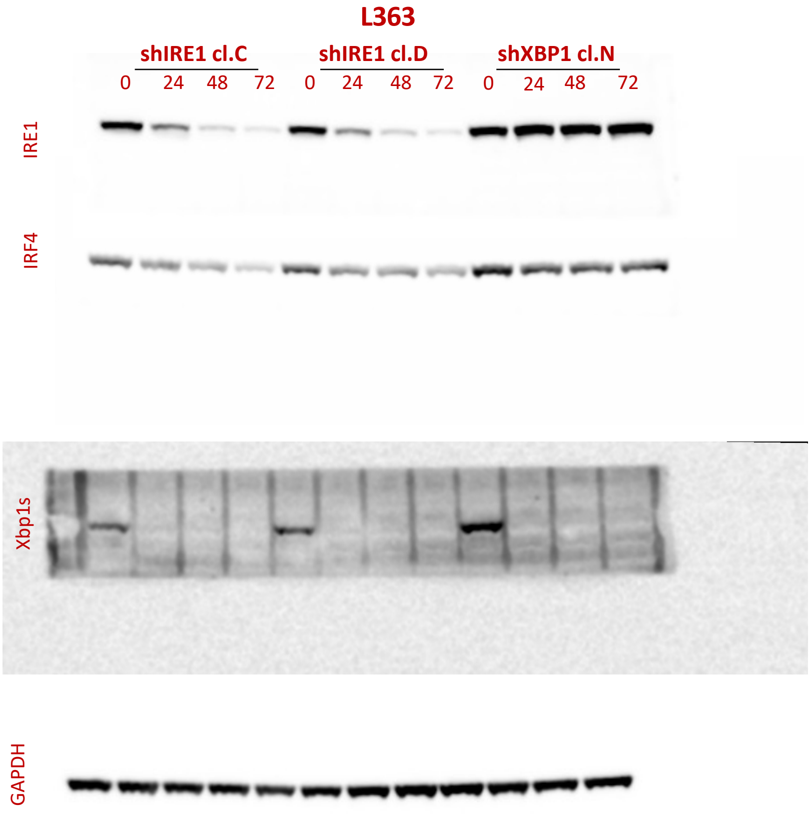
L363
shIRE1 cl.C
shIRE1 cl.D
shXBP1 cl.N
0 24 48 72 0 24 48 72 0 24 48 72
IRE1
IRF4
Xbp1s
GAPDH

S1D

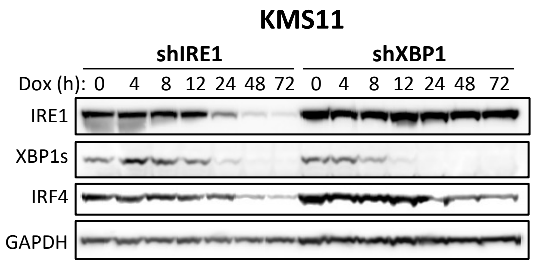
KMS11
shIRE1
shXBP1
Dox (h): 0 4 8 12 24 48 72 0 4 8 12 24 48 72
IRE1
XBP1s
IRF4
GAPDH

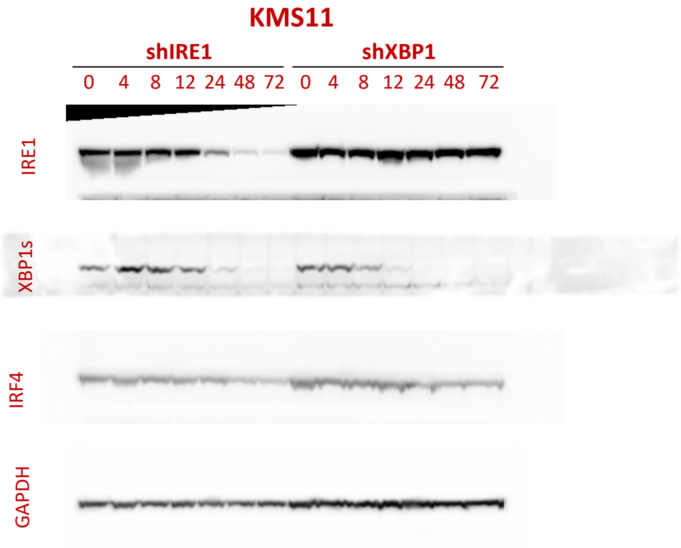
KMS11
shIRE1
shXBP1
0 4 8 12 24 48 72 0 4 8 12 24 48 72
IRE1
XBP1s
IRF4
GAPDH

S1F

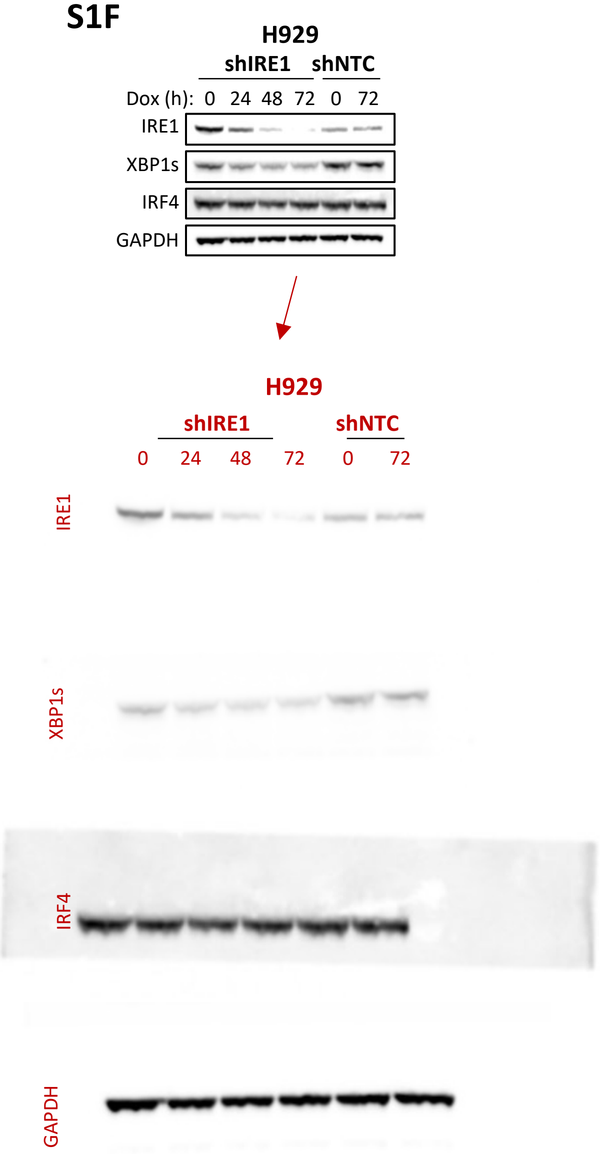
H929
shIRE1 shNTC
Dox (h): 0 24 48 72 0 72
IRE1
XBP1s
IRF4
GAPDH
H929
shIRE1
shNTC
0 24 48 72 0 72
IRE1
XBP1s
IRF4
GAPDH

S1H

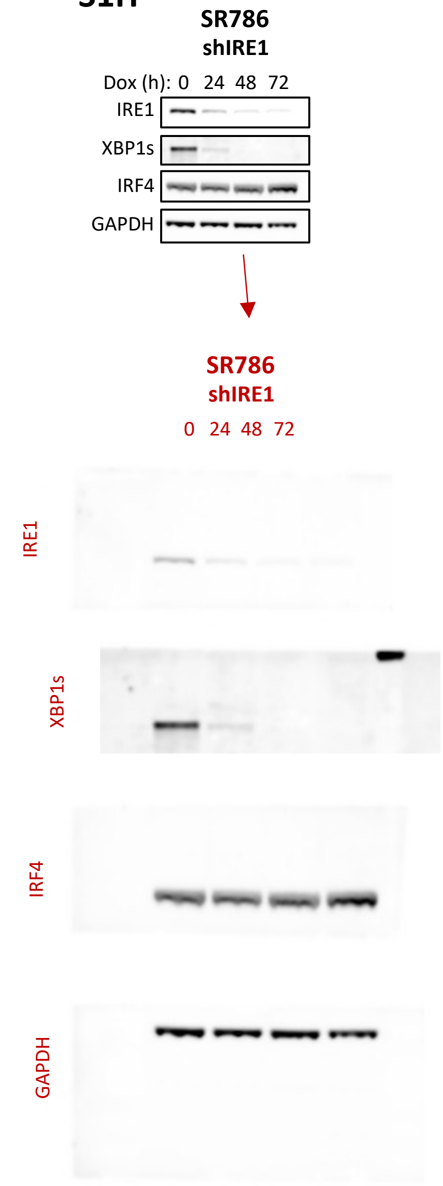
SR786
shIRE1
Dox (h): 0 24 48 72
IRE1
XBP1s
IRF4
GAPDH
SR786
shIRE1
0 24 48 72
IRE1
XBP1s
IRF4
GAPDH

2B

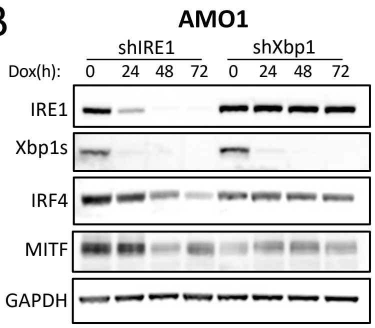
AMO1
shIRE1
shXbp1
Dox(h):
0 24 48 72 0 24 48 72
IRE1
Xbp1s
IRF4
MITF
GAPDH

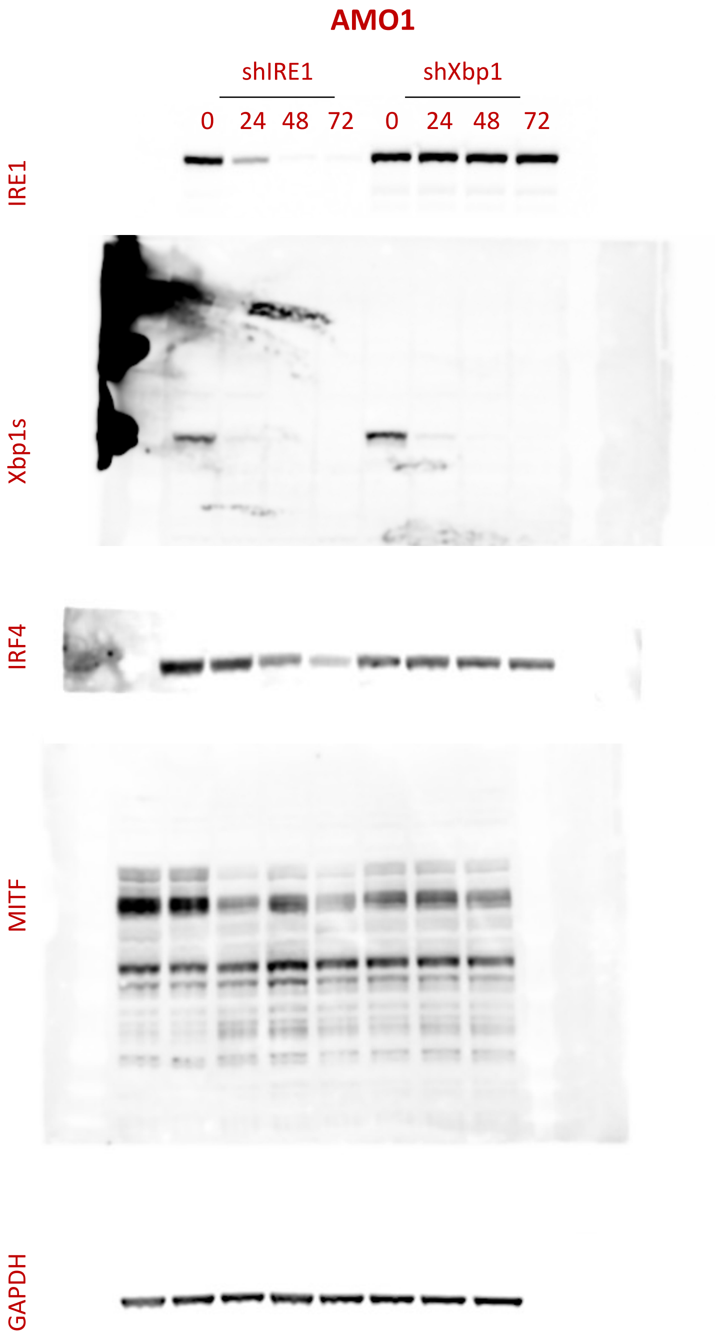
AMO1
shIRE1
shXbp1
0 24 48 72 0 24 48 72
IRE1
Xbp1s
IRF4
MITF
GAPDH

2D

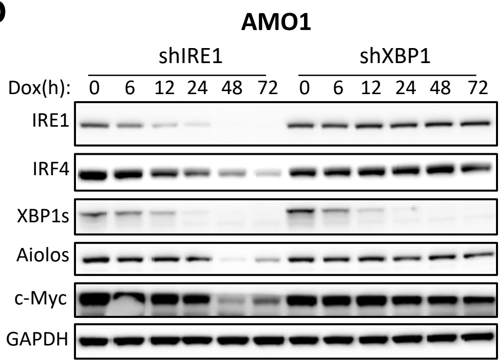

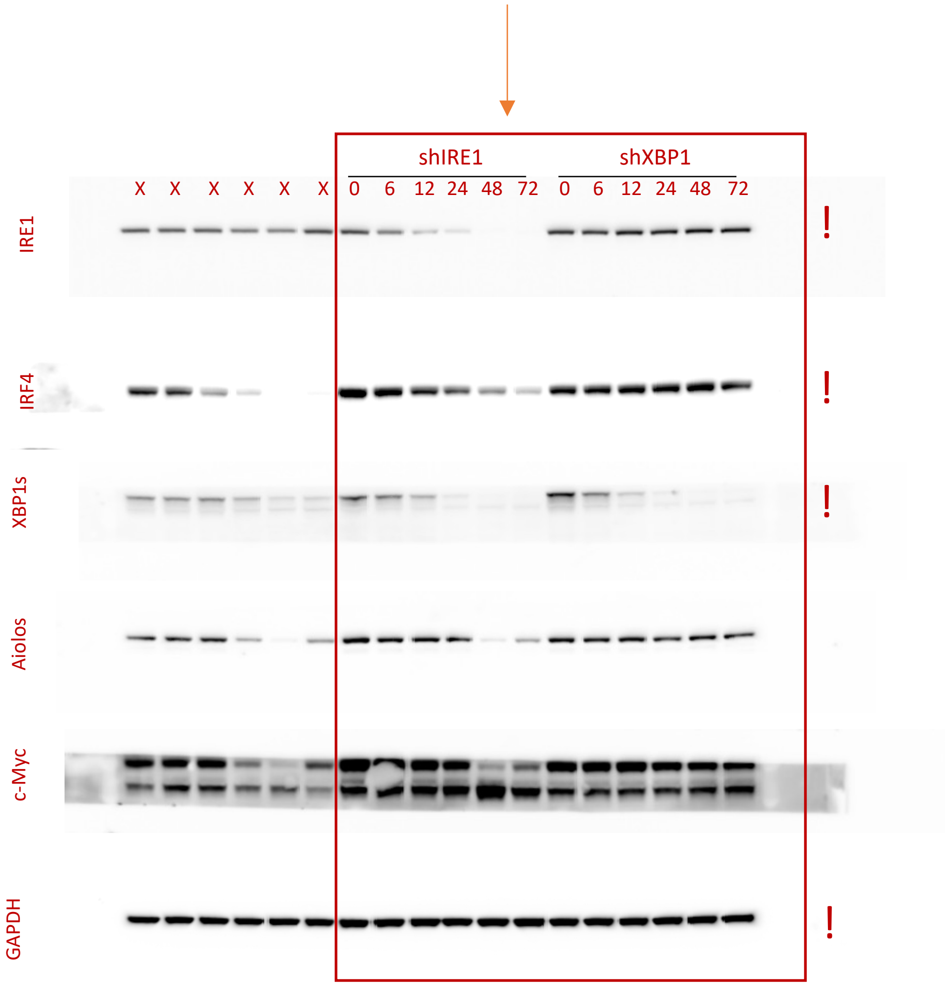

! Blots repeated in Fig S3C and S4E

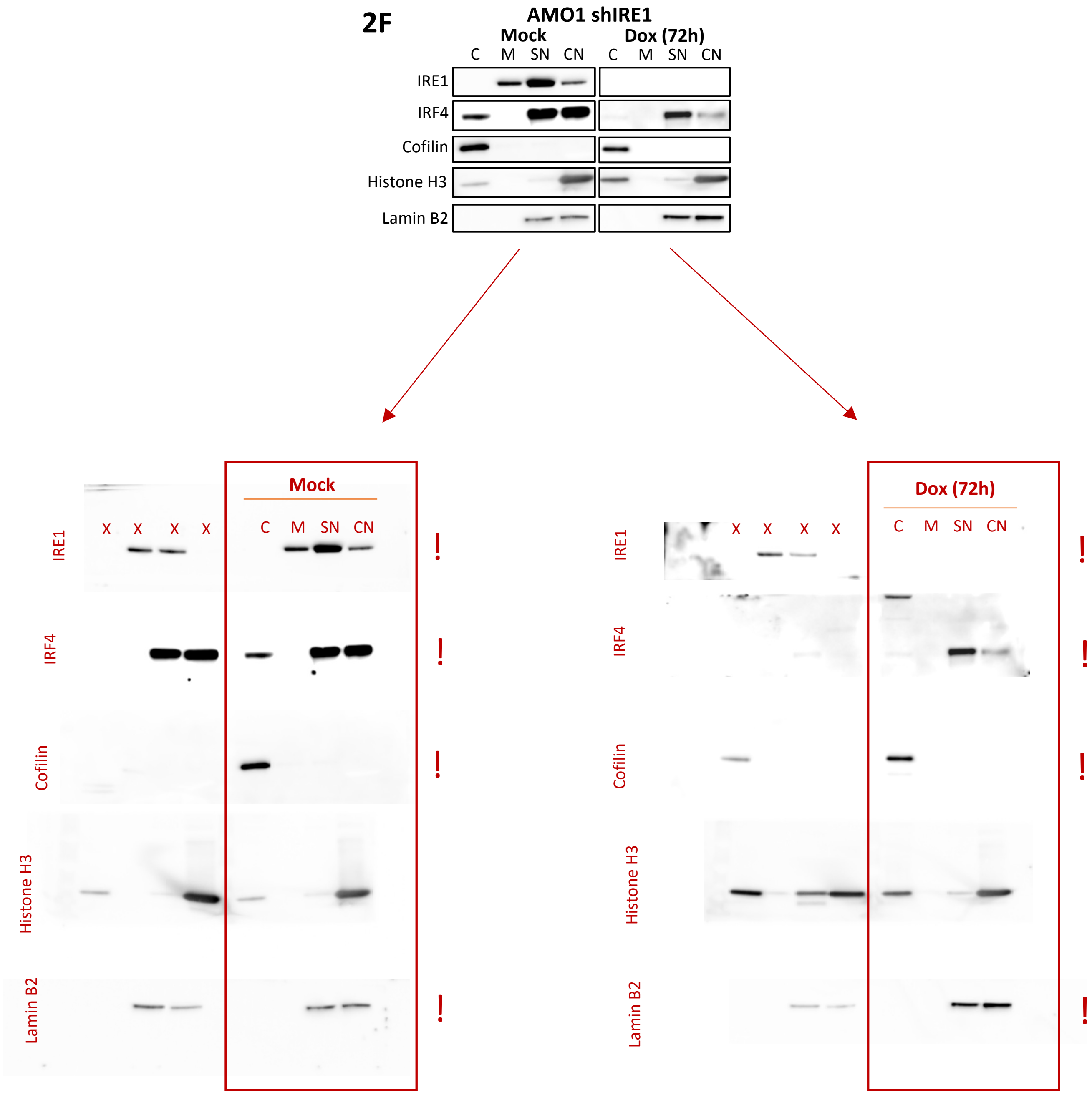

! Control blots repeated in Fig. 3G

S2B

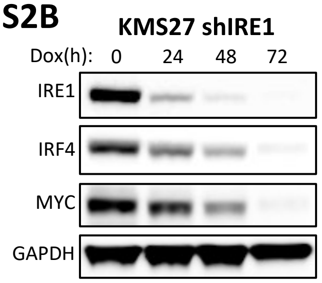
KMS27 shIRE1
Dox(h): 0 24 48 72
IRE1
IRF4
MYC
GAPDH

S2D

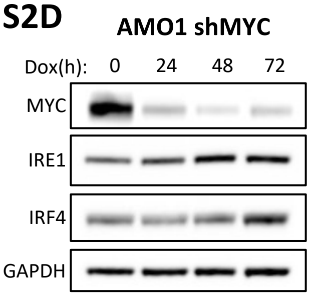
AMO1 shMYC
Dox(h): 0 24 48 72
MYC
IRE1
IRF4
GAPDH

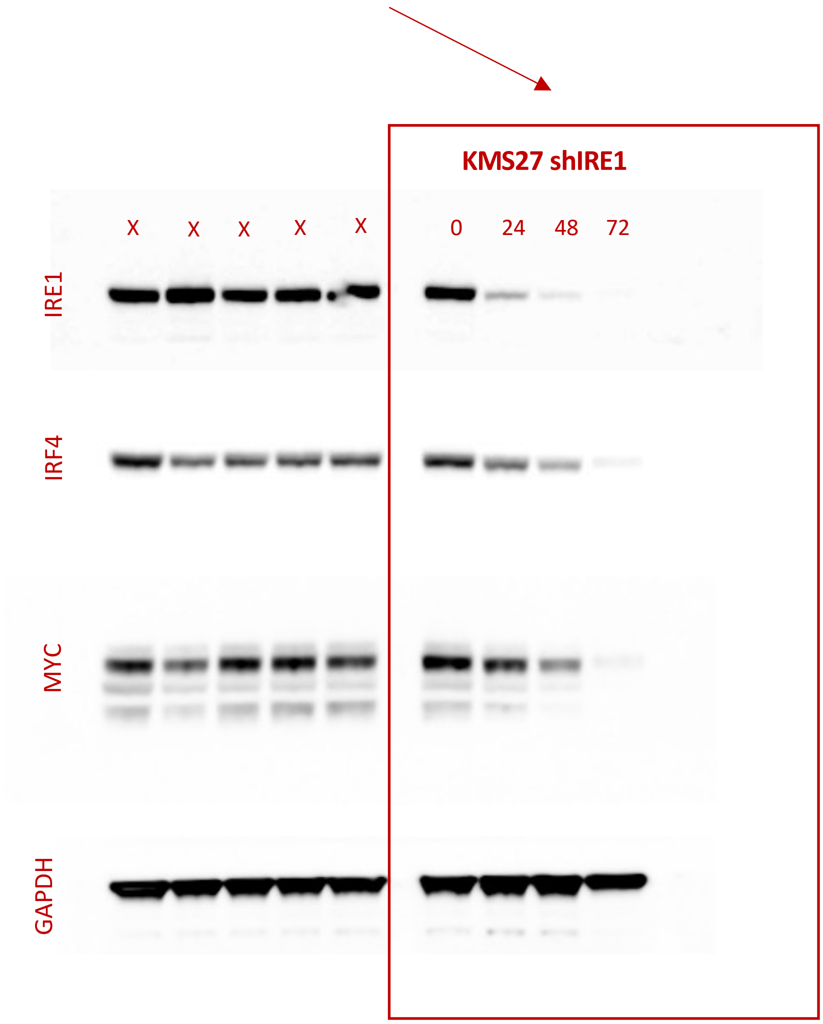
KMS27 shIRE1
X X X X X 0 24 48 72
IRE1
IRF4
MYC
GAPDH

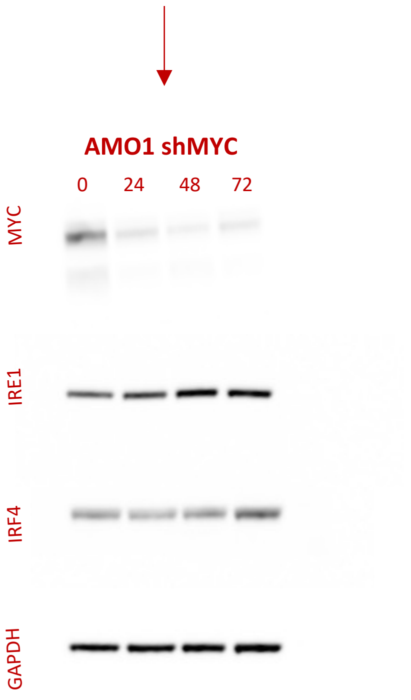
AMO1 shMYC
0 24 48 72
MYC
IRE1
IRF4
GAPDH

S2C

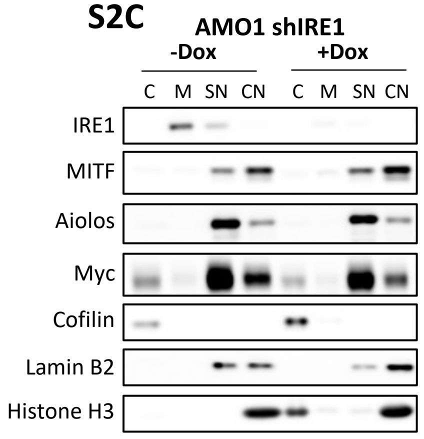
AMO1 shIRE1
-Dox
+Dox
C M SN CN C M SN CN
IRE1
MITF
Aiolos
Myc
Cofilin
Lamin B2
Histone H3

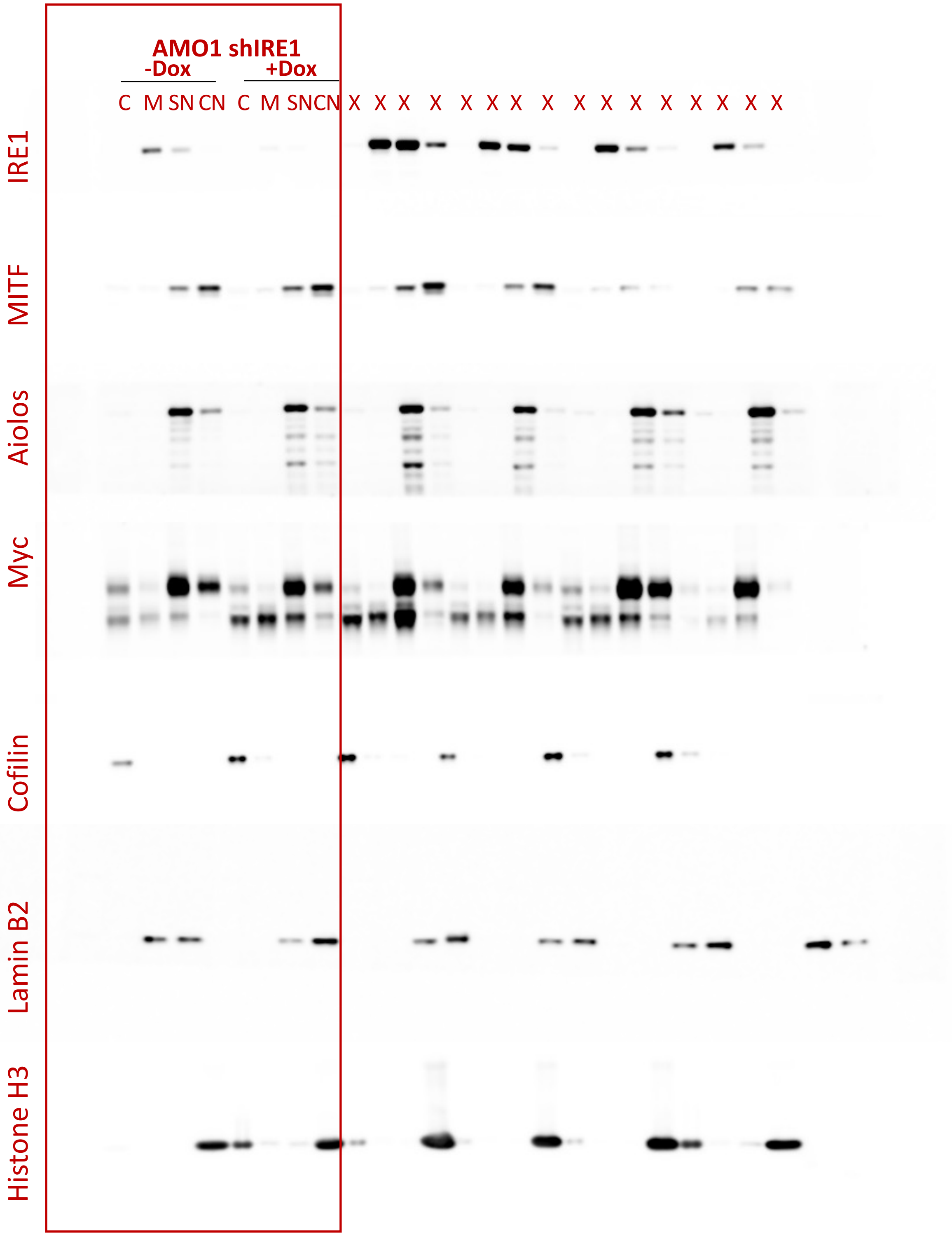
AMO1 shIRE1
-Dox
+Dox
C M SN CN C M SNCN X X X X X X X X X X X X X X X X
IRE1
MITF
Aiolos
Myc
Cofilin
Lamin B2
Histone H3

S2E

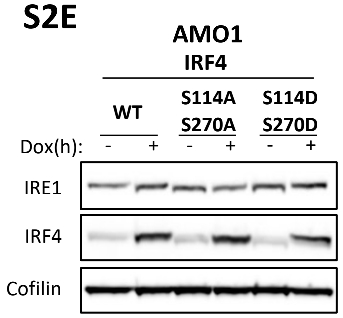
AMO1
IRF4
WT
S114A
S270A
S114D
S270D
Dox(h):
- + - + - +
IRE1
IRF4
Cofilin

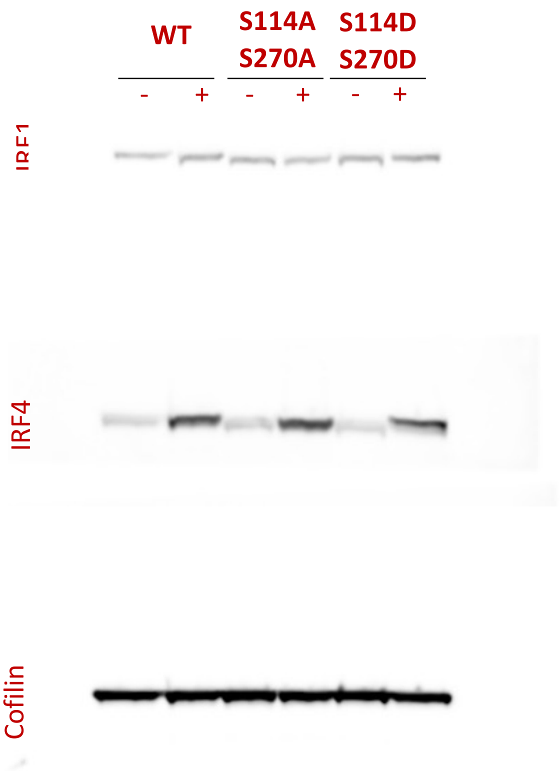
WT
S114A
S270A
S114D
S270D
- + - + - +
IRF4
Cofilin

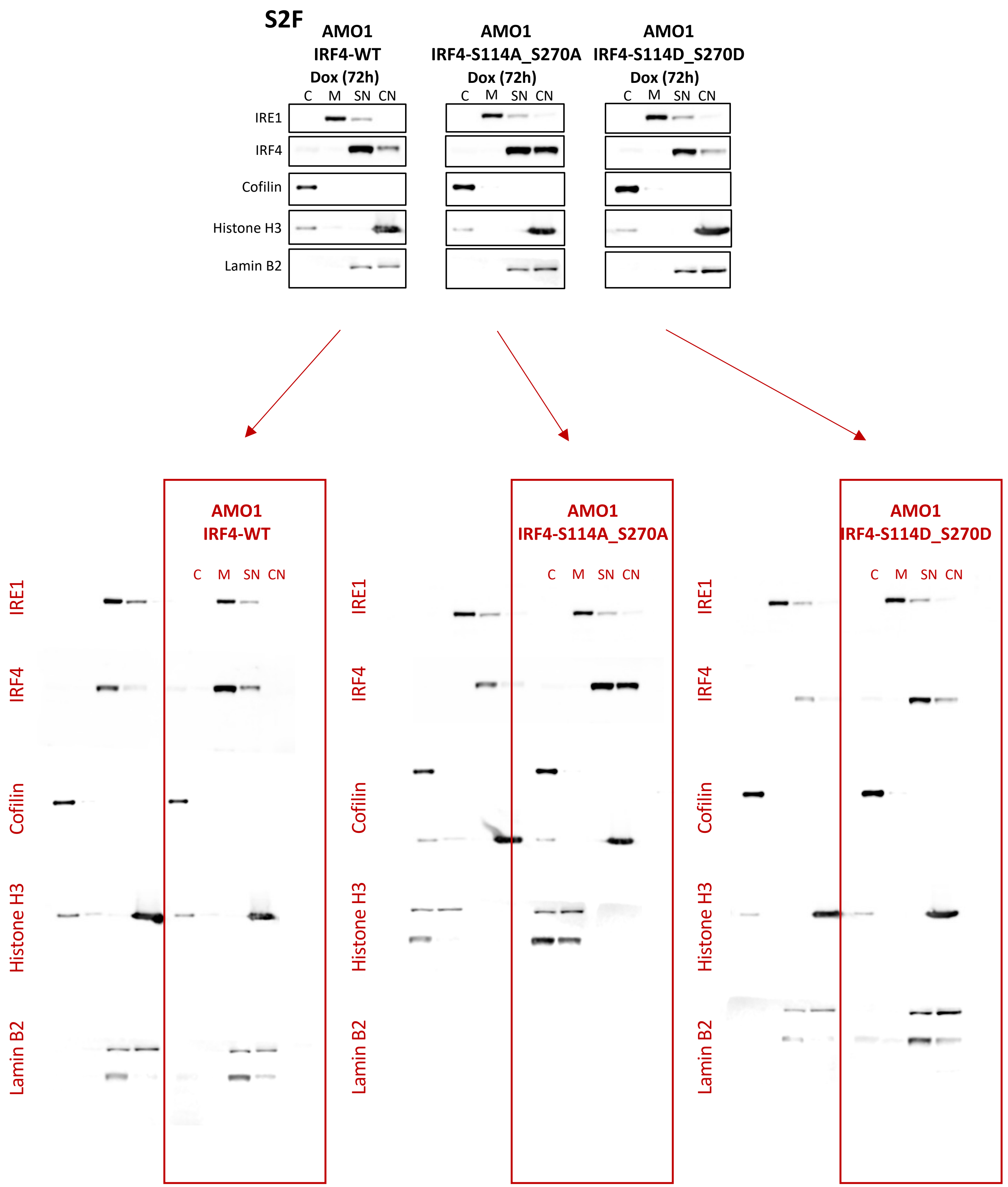
S2F
AMO1
IRF4-WT
Dox (72h)
C M SN CN
AMO1
IRF4-S114A_S270A
Dox (72h)
C M SN CN
AMO1
IRF4-S114D_S270D
Dox (72h)
C M SN CN
IRE1
IRF4
Cofilin
Histone H3
Lamin B2
AMO1
IRF4-WT
C M SN CN
AMO1
IRF4-S114A_S270A
C M SN CN
AMO1
IRF4-S114D_S270D
C M SN CN
IRE1
IRF4
Cofilin
Histone H3
Lamin B2

3F

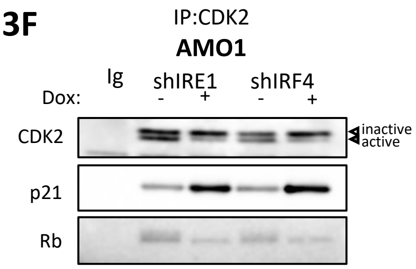
IP:CDK2
AMO1
Ig
shIRE1
shIRF4
Dox:
-
+
-
+
CDK2
inactive
active
p21
Rb

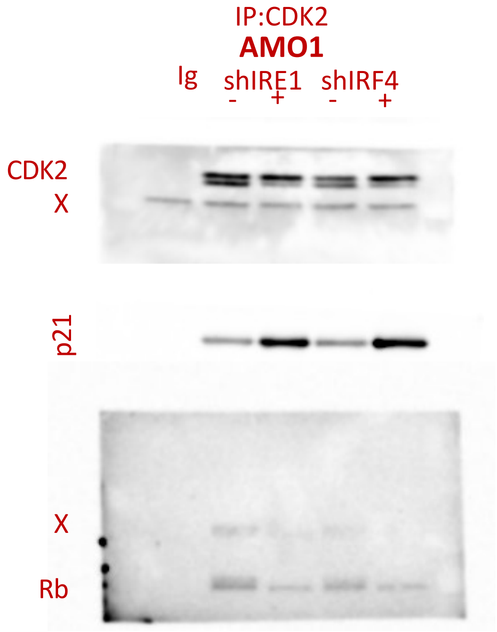
IP:CDK2
AMO1
Ig
shIRE1
shIRF4
-
+
-
+
CDK2
X
p21
X
Rb

3G

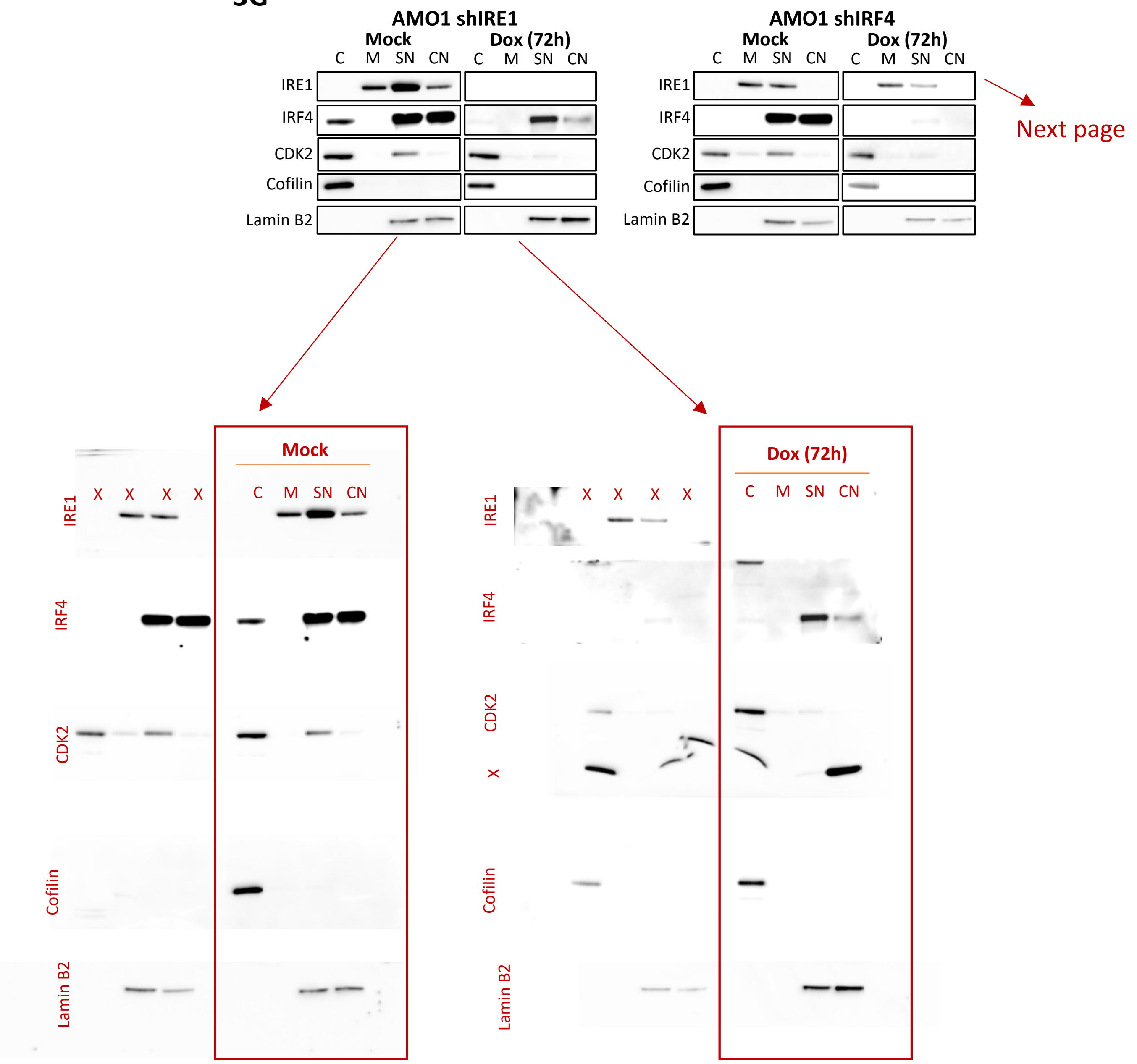

! Control blots repeated in Fig. 2F

3G

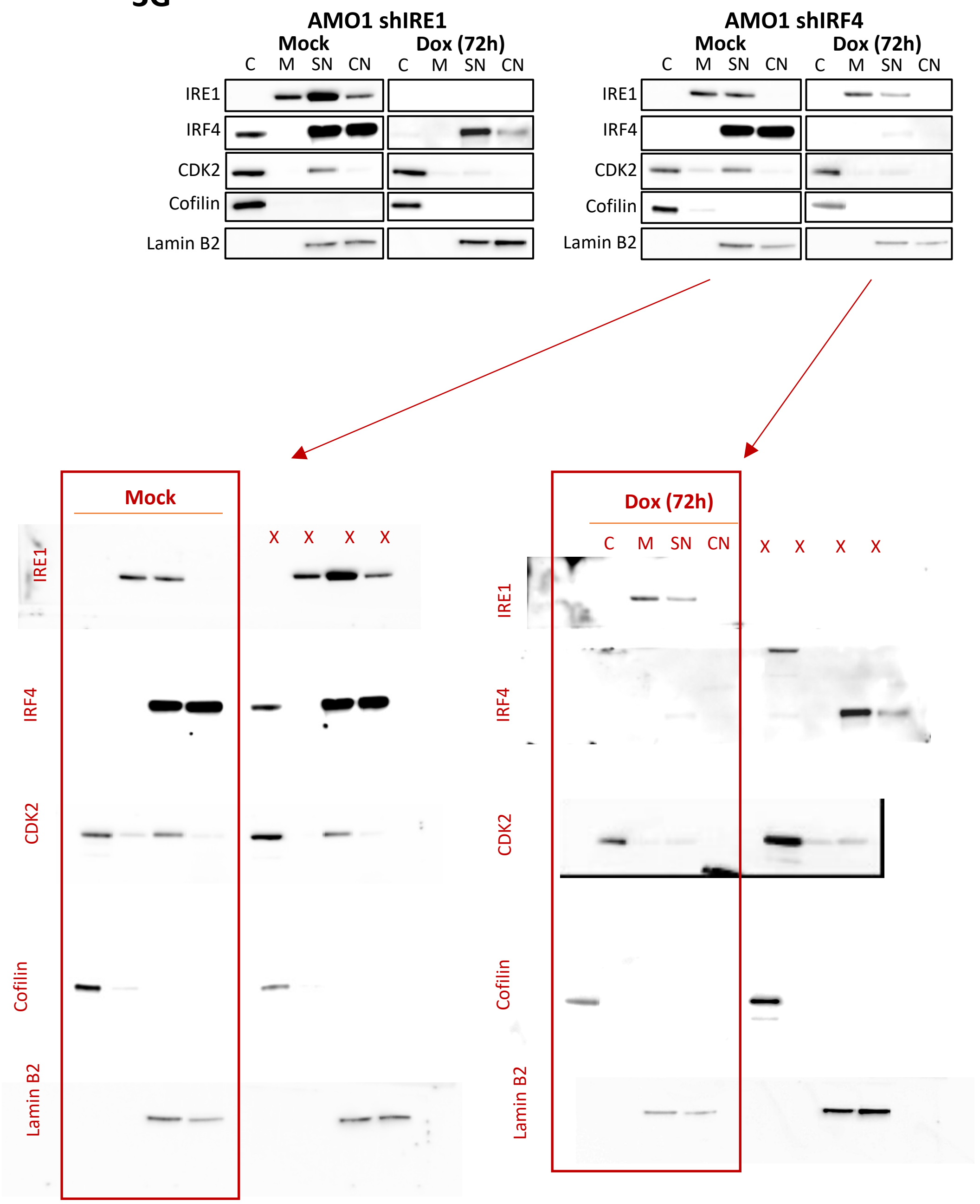

! Control blots repeated in Fig. 2F

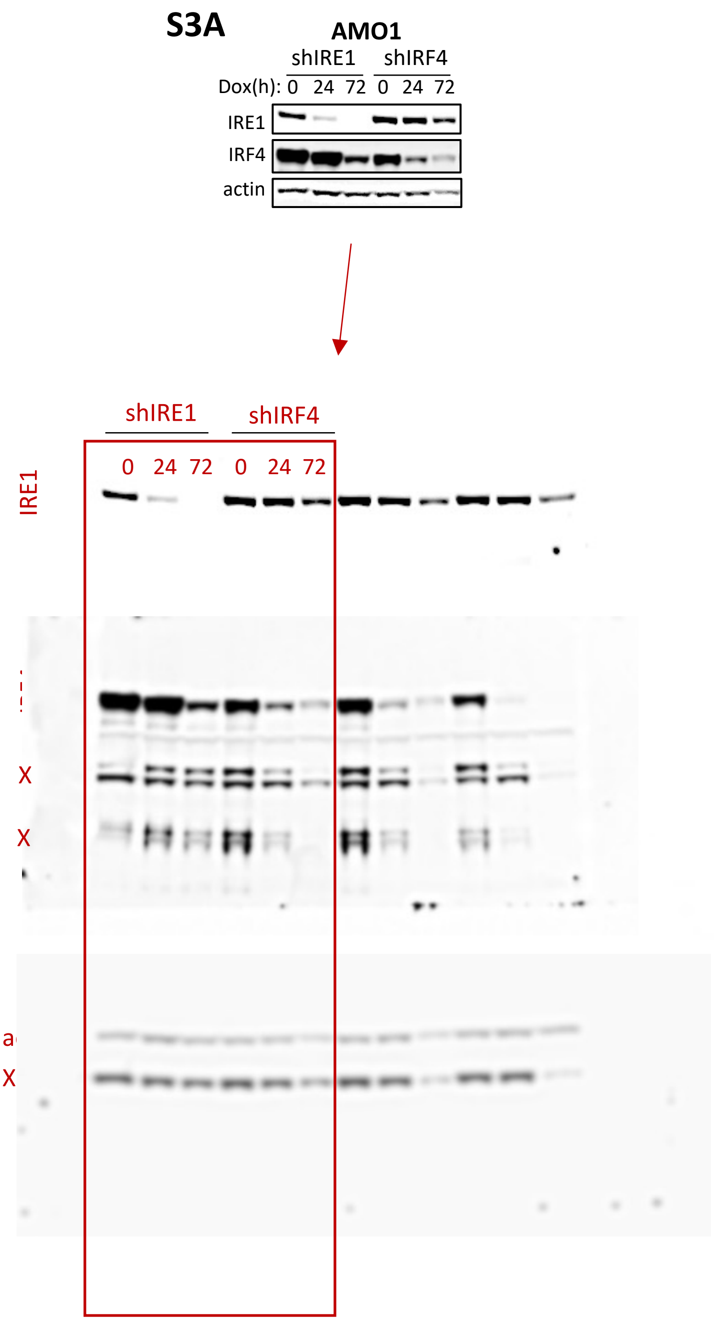
S3A
AMO1
shIRE1
shIRF4
Dox(h): 0 24 72 0 24 72
IRE1
IRF4
actin
shIRE1
shIRF4
0 24 72 0 24 72
IRE1
X
X
X

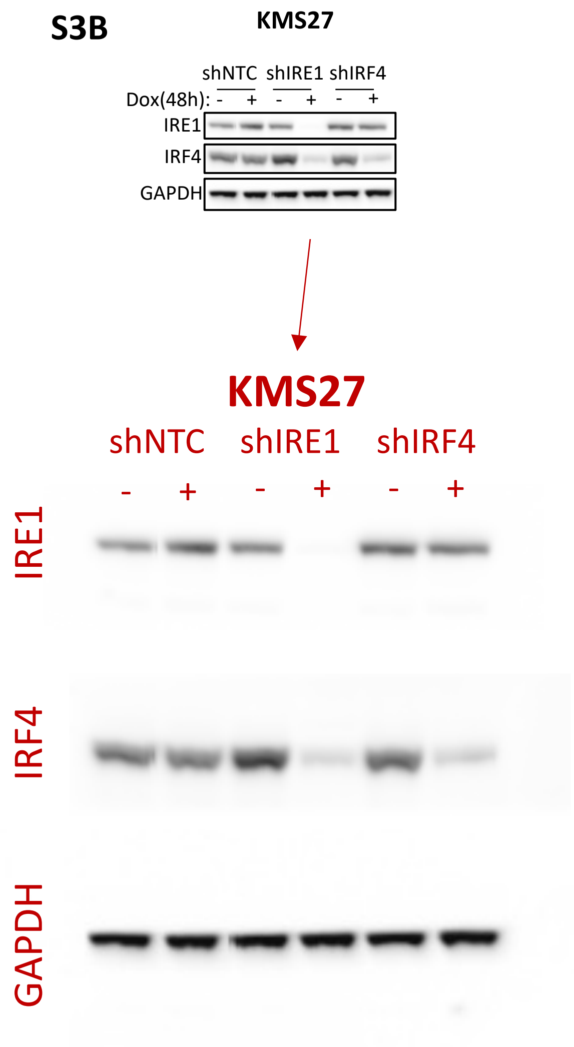
S3B
KMS27
shNTC shIRE1 shIRF4
Dox(48h): - + - + - +
IRE1
IRF4
GAPDH
KMS27
shNTC
shIRE1
shIRF4
- + - + - +
IRE1
IRF4
GAPDH

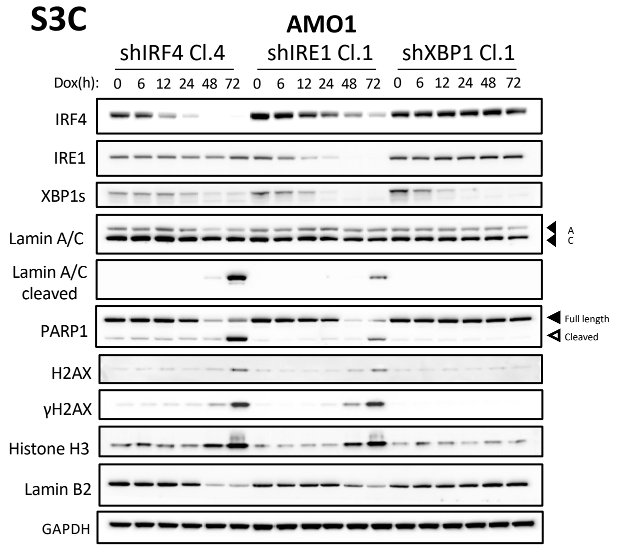

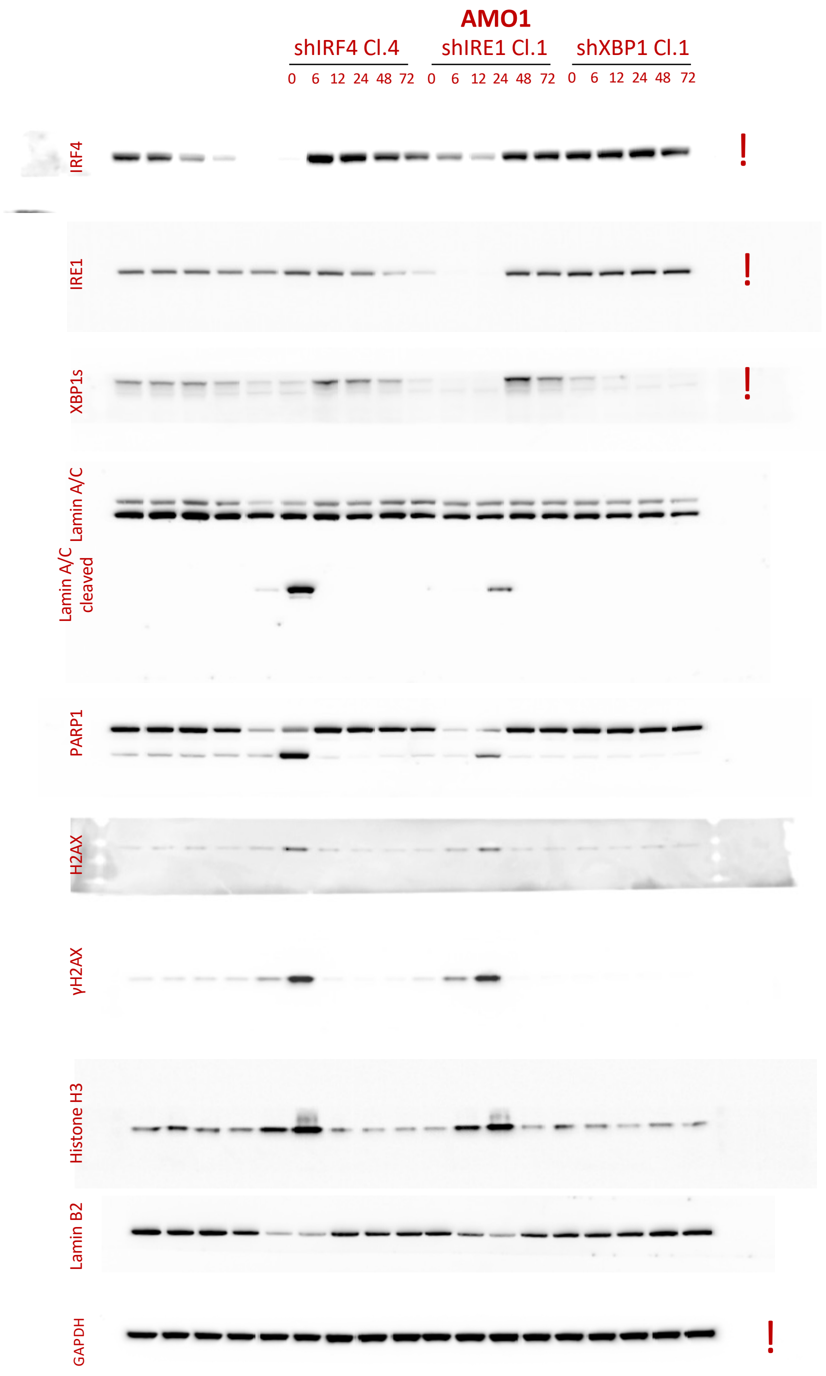

! Control blots repeated in Fig 2D and S4E

S3F

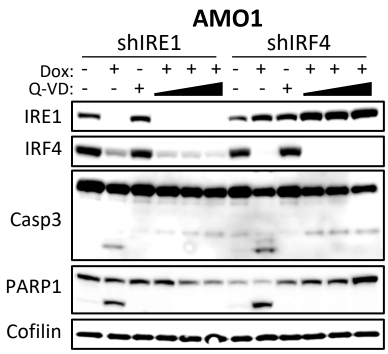

AMO1
shIRE1
shIRF4
Dox: - + - + + + - + - + + +
Q-VD: - - + - - +
IRE1
IRF4
Casp3
PARP1
Cofilin

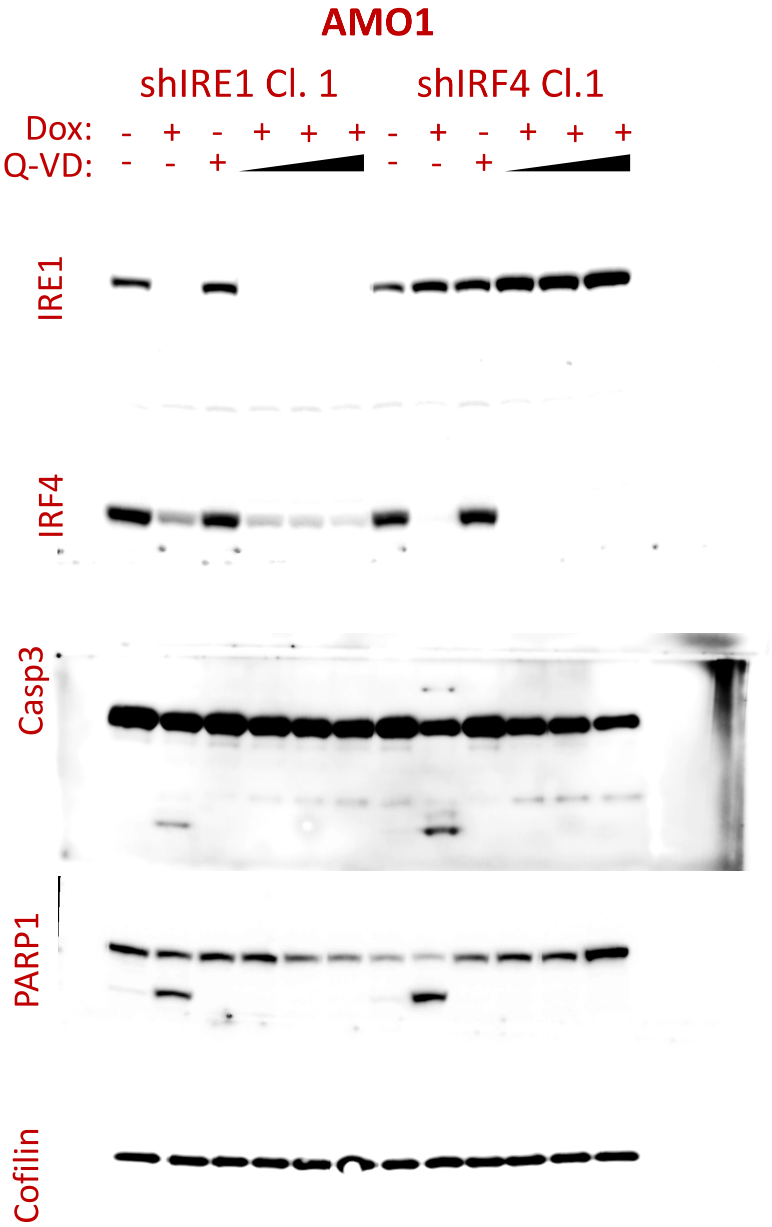

AMO1
shIRE1 Cl. 1
shIRF4 Cl.1
Dox: - + - + + + - + - + + +
Q-VD: - - + - - +
IRE1
IRF4
Casp3
PARP1
Cofilin

S3N

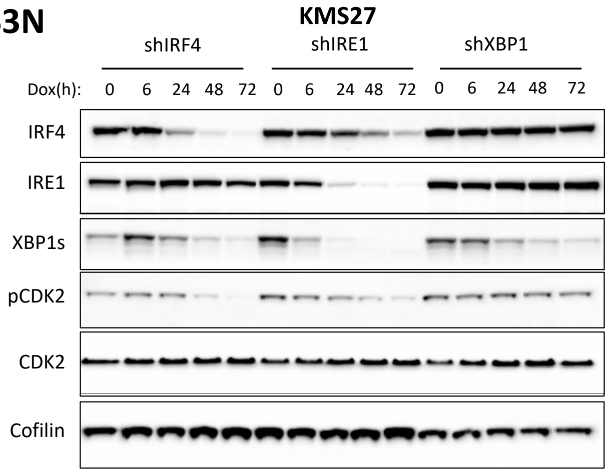
KMS27
shIRF4
shIRE1
shXBP1
Dox(h): 0 6 24 48 72 0 6 24 48 72 0 6 24 48 72
IRF4
IRE1
XBP1s
pCDK2
CDK2
Cofilin

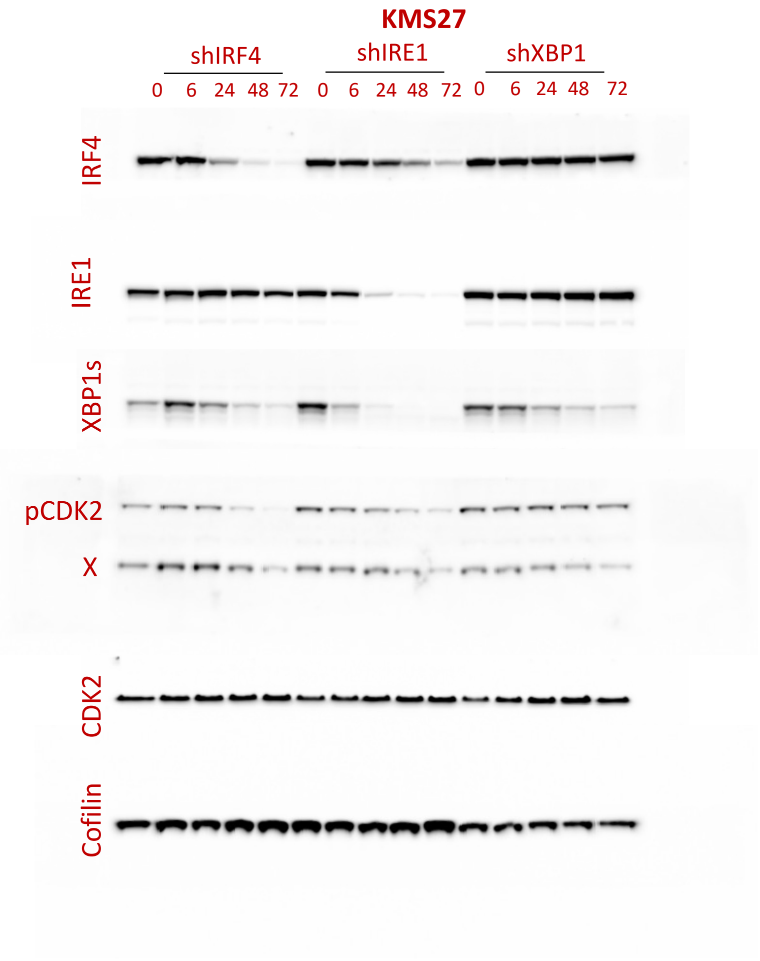
KMS27
shIRF4
shIRE1
shXBP1
0 6 24 48 72 0 6 24 48 72 0 6 24 48 72
IRF4
IRE1
XBP1s
pCDK2
X
CDK2
Cofilin

S3O

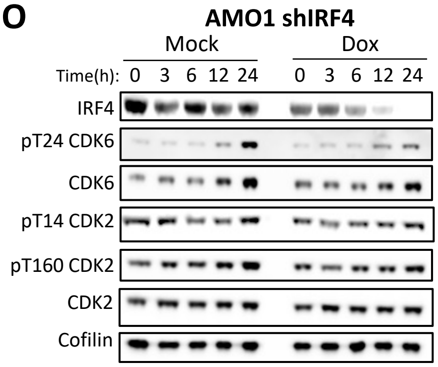
AMO1 shIRF4
Mock
Dox
Time(h): 0 3 6 12 24 0 3 6 12 24
IRF4
pT24 CDK6
CDK6
pT14 CDK2
pT160 CDK2
CDK2
Cofilin

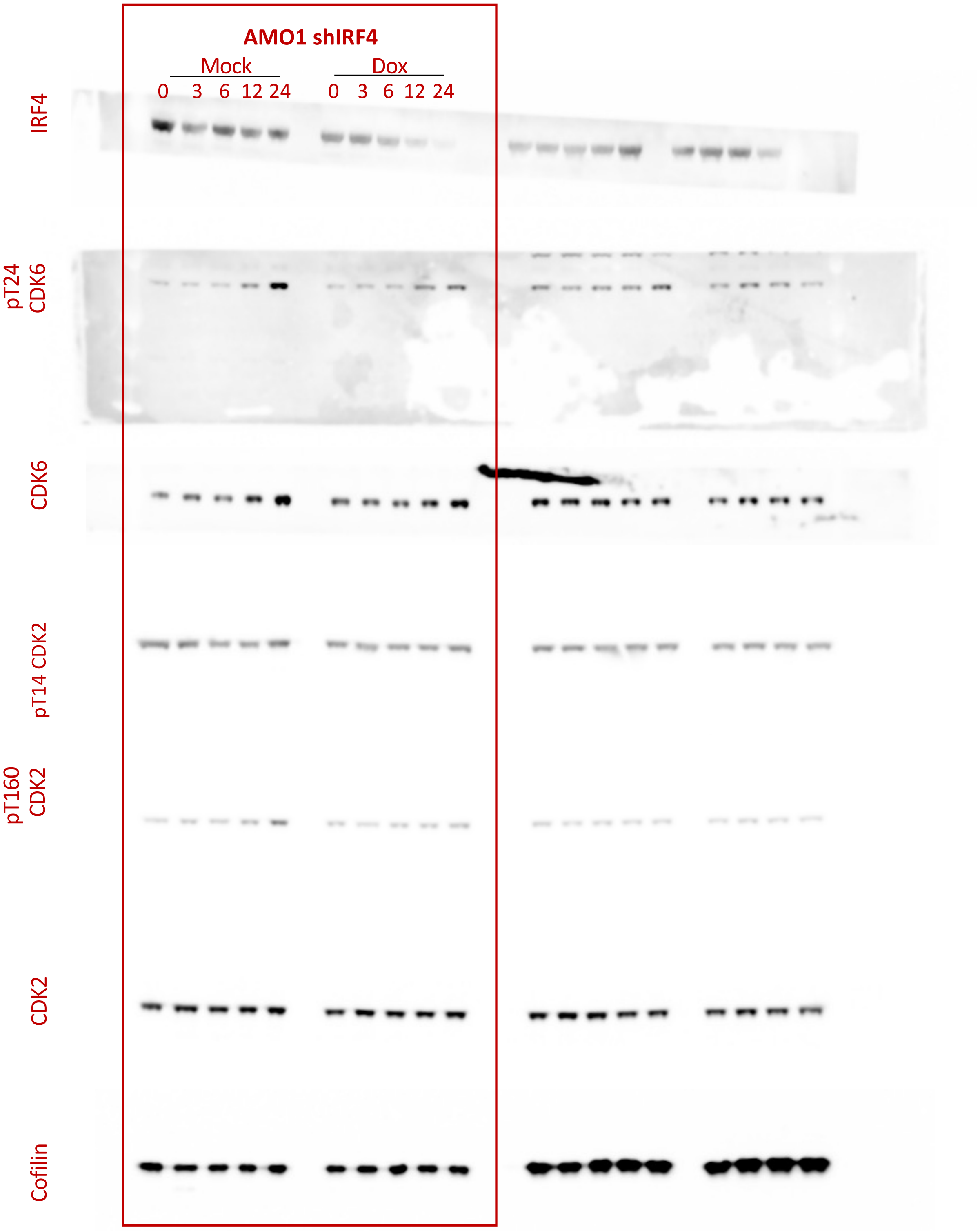
AMO1 shIRF4
Mock
Dox
0 3 6 12 24 0 3 6 12 24
IRF4
pT24 CDK6
CDK6
pT14 CDK2
pT160 CDK2
CDK2
Cofilin

S3P

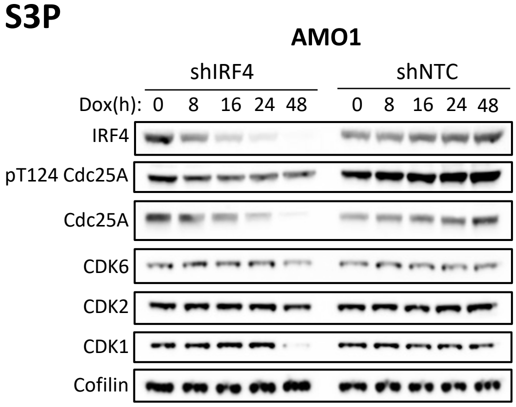
AMO1
shIRF4
shNTC
Dox(h): 0 8 16 24 48 0 8 16 24 48
IRF4
pT124 Cdc25A
Cdc25A
CDK6
CDK2
CDK1
Cofilin

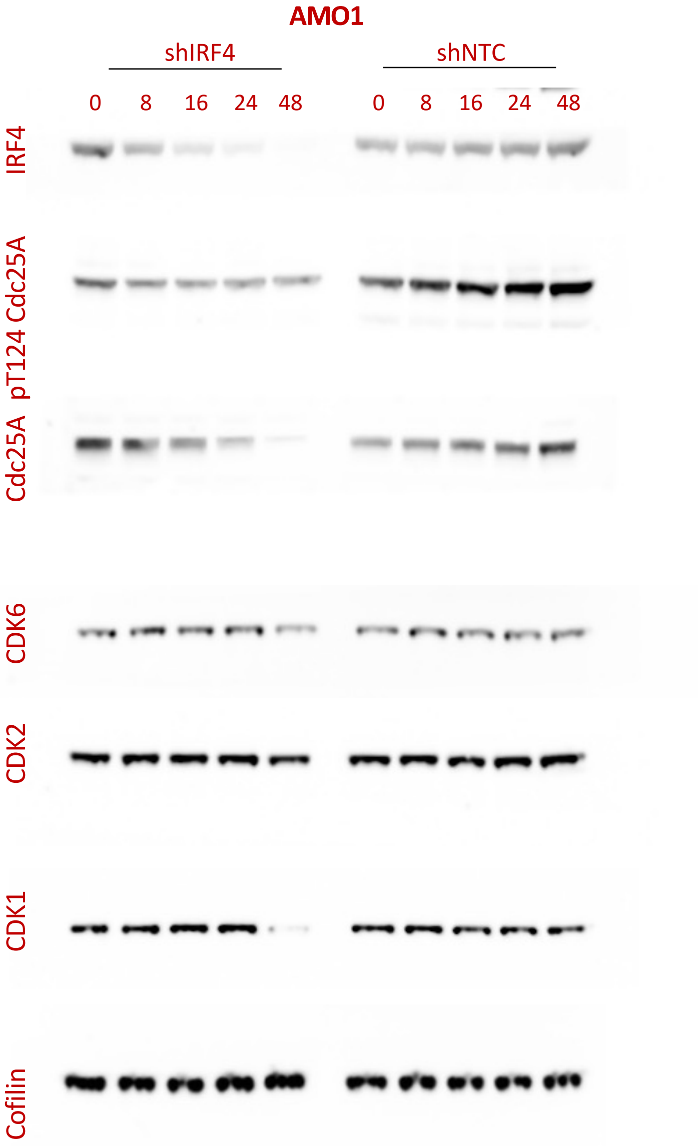
AMO1
shIRF4
shNTC
0 8 16 24 48 0 8 16 24 48
IRF4
pT124 Cdc25A
Cdc25A
CDK6
CDK2
CDK1
Cofilin

4D

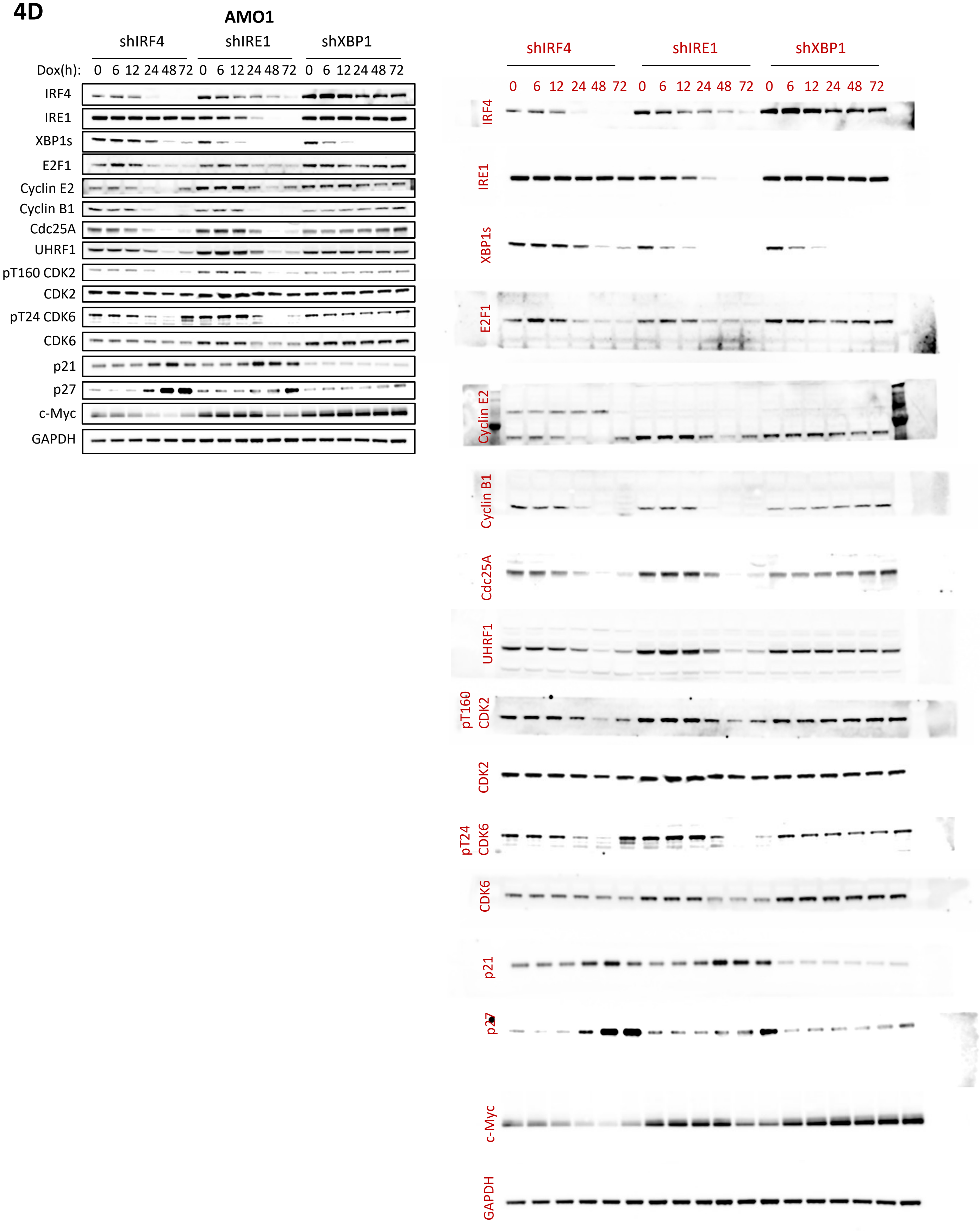

AMO1
shIRF4
shIRE1
shXBP1
Dox(h): 0 6 12 24 48 72 0 6 12 24 48 72 0 6 12 24 48 72
IRF4
IRE1
XBP1s
E2F1
Cyclin E2
Cyclin B1
Cdc25A
UHRF1
pT160 CDK2
CDK2
pT24 CDK6
CDK6
p21
p27
c-Myc
GAPDH

S4E

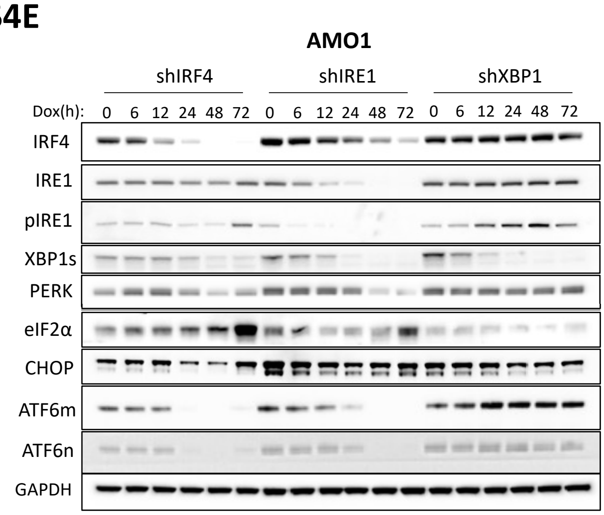

! Control blots repeated in Fig 2D and S3C

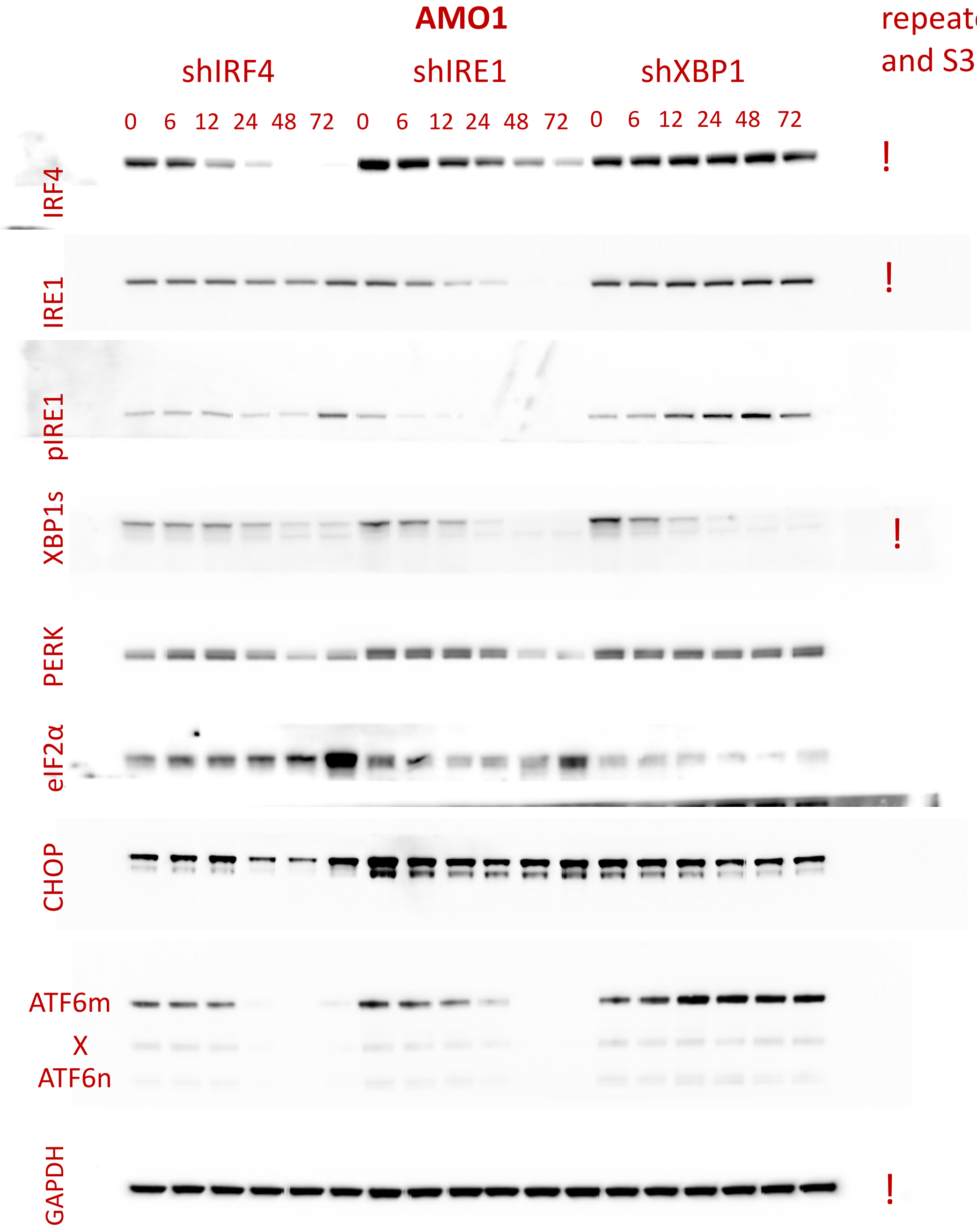

5E

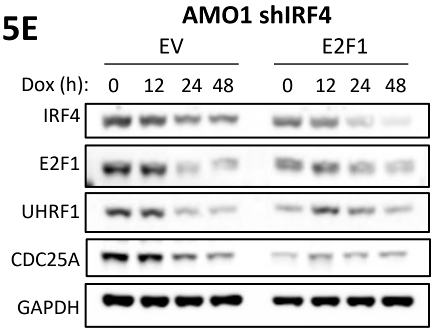
AMO1 shIRF4
EV
E2F1
Dox (h): 0 12 24 48 0 12 24 48
IRF4
E2F1
UHRF1
CDC25A
GAPDH

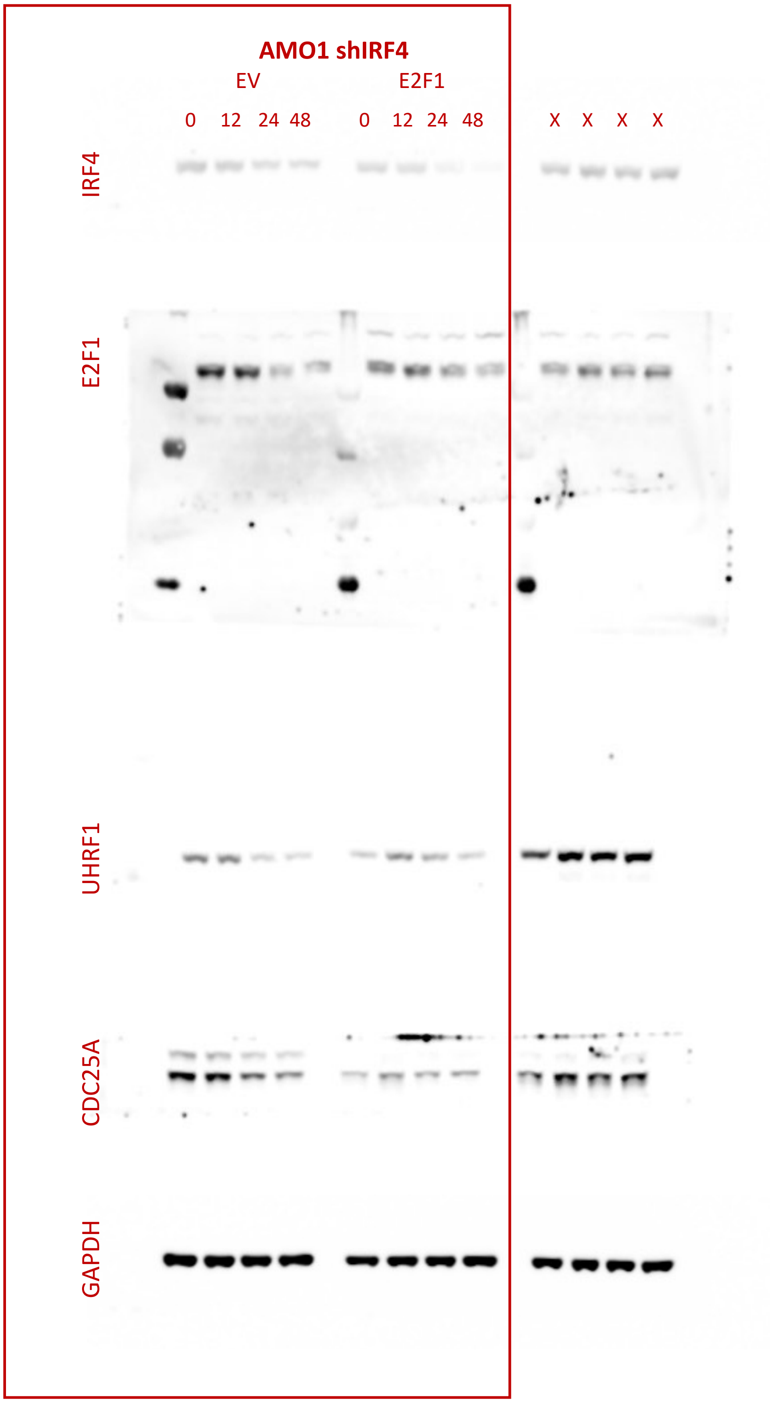
AMO1 shIRF4
EV
E2F1
0 12 24 48 0 12 24 48
X X X X
IRF4
E2F1
UHRF1
CDC25A
GAPDH

6A

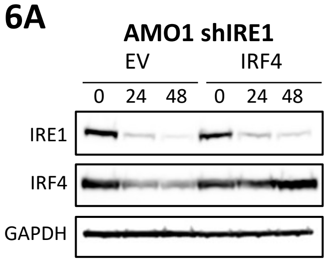
AMO1 shIRE1
EV
IRF4
0 24 48 0 24 48
IRE1
IRF4
GAPDH

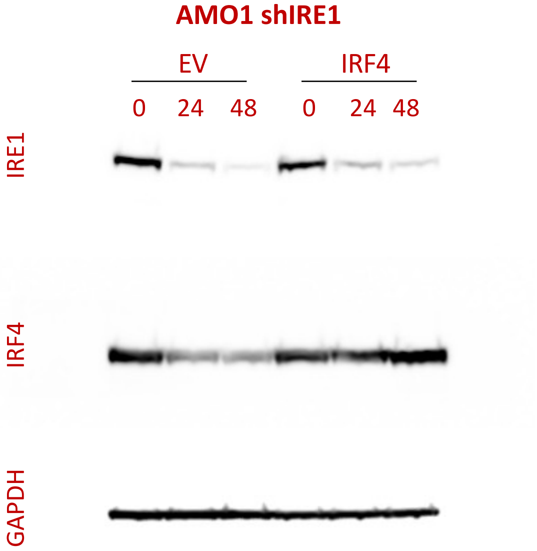
AMO1 shIRE1
EV
IRF4
0 24 48 0 24 48
IRE1
IRF4
GAPDH

6G

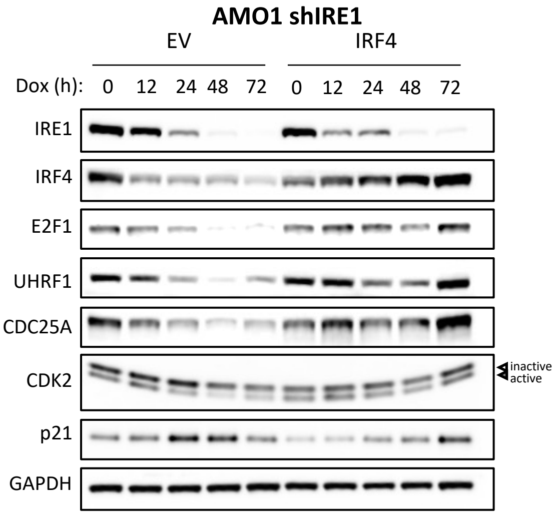
AMO1 shIRE1
EV
IRF4
Dox (h): 0 12 24 48 72 0 12 24 48 72
IRE1
IRF4
E2F1
UHRF1
CDC25A
CDK2
inactive
active
p21
GAPDH

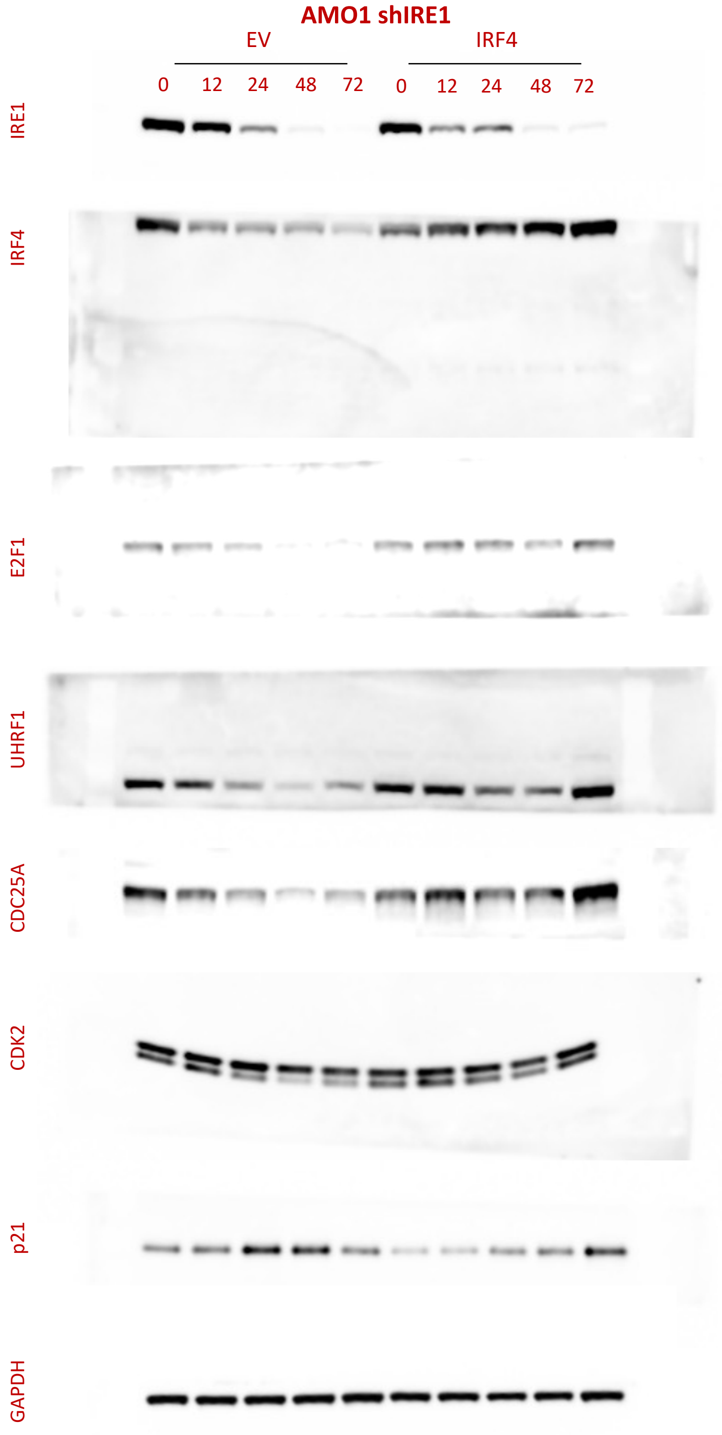
AMO1 shIRE1
EV
IRF4
0 12 24 48 72 0 12 24 48 72
IRE1
IRF4
E2F1
UHRF1
CDC25A
CDK2
p21
GAPDH

6K

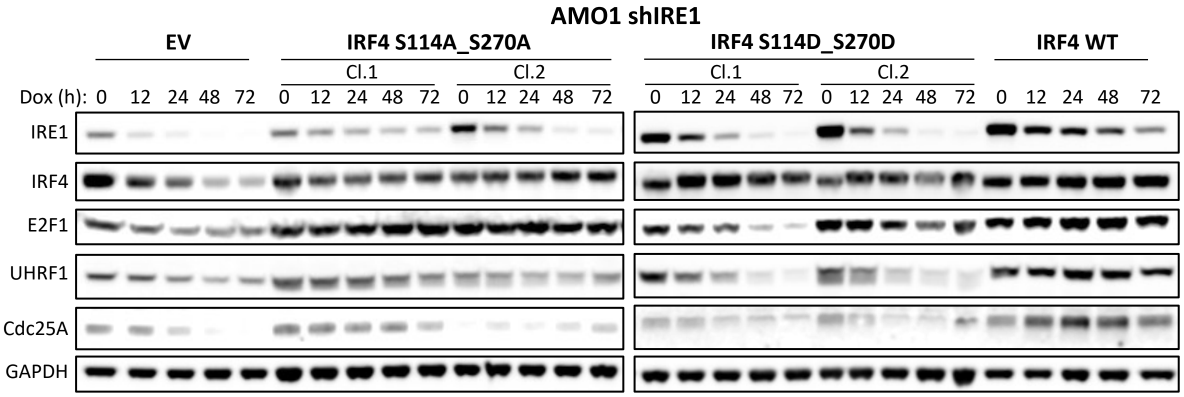
AMO1 shIRE1
EV
IRF4 S114A_S270A
Cl.1
Cl.2
IRF4 S114D_S270D
Cl.1
Cl.2
IRF4 WT
Dox (h): 0 12 24 48 72 0 12 24 48 72 0 12 24 48 72 0 12 24 48 72 0 12 24 48 72 0 12 24 48 72
IRE1
IRF4
E2F1
UHRF1
Cdc25A
GAPDH

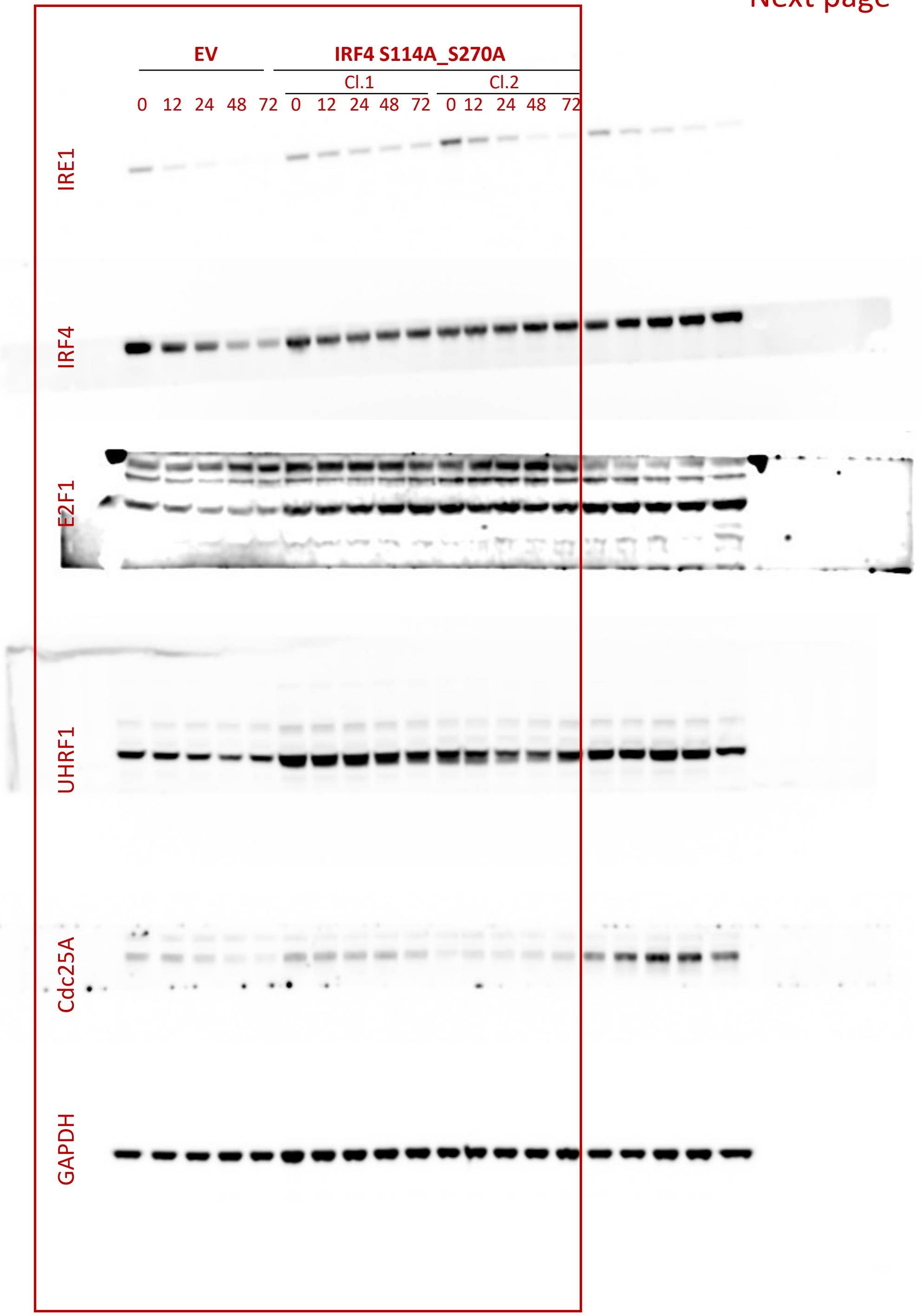
EV
IRF4 S114A_S270A
Cl.1
Cl.2
0 12 24 48 72 0 12 24 48 72 0 12 24 48 72
IRE1
IRF4
E2F1
UHRF1
Cdc25A
GAPDH

Next page

6K

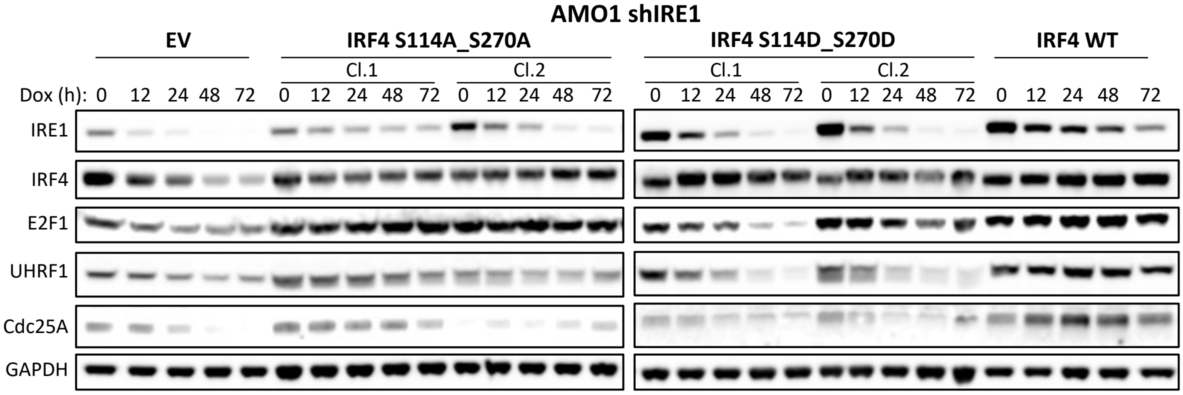

AMO1 shIRE1
EV
IRF4 S114A_S270A
IRF4 S114D_S270D
IRF4 WT
Cl.1
Cl.2
Cl.1
Cl.2
Dox (h): 0 12 24 48 72 0 12 24 48 72 0 12 24 48 72 0 12 24 48 72 0 12 24 48 72 0 12 24 48 72
IRE1
IRF4
E2F1
UHRF1
Cdc25A
GAPDH

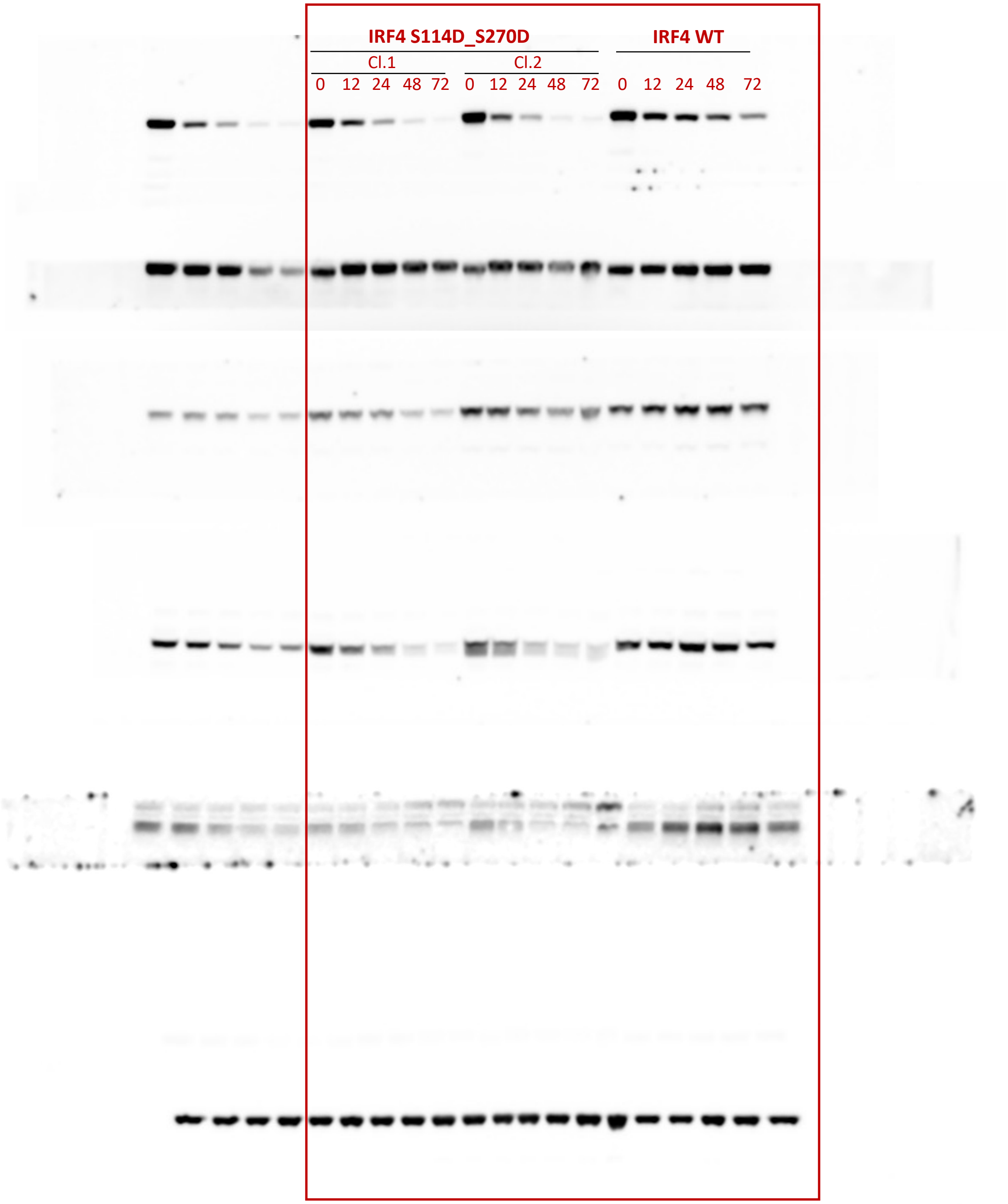

IRF4 S114D_S270D
IRF4 WT
Cl.1
Cl.2
0 12 24 48 72 0 12 24 48 72 0 12 24 48 72

S6D

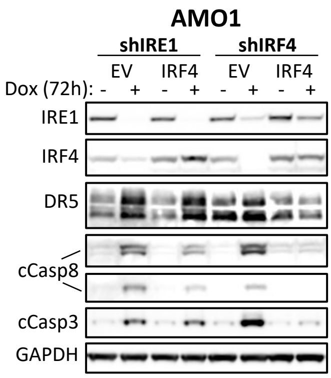
AMO1
shIRE1
shIRF4
EV
IRF4
EV
IRF4
Dox (72h): - + - + - + - +
IRE1
IRF4
DR5
cCasp8
cCasp3
GAPDH

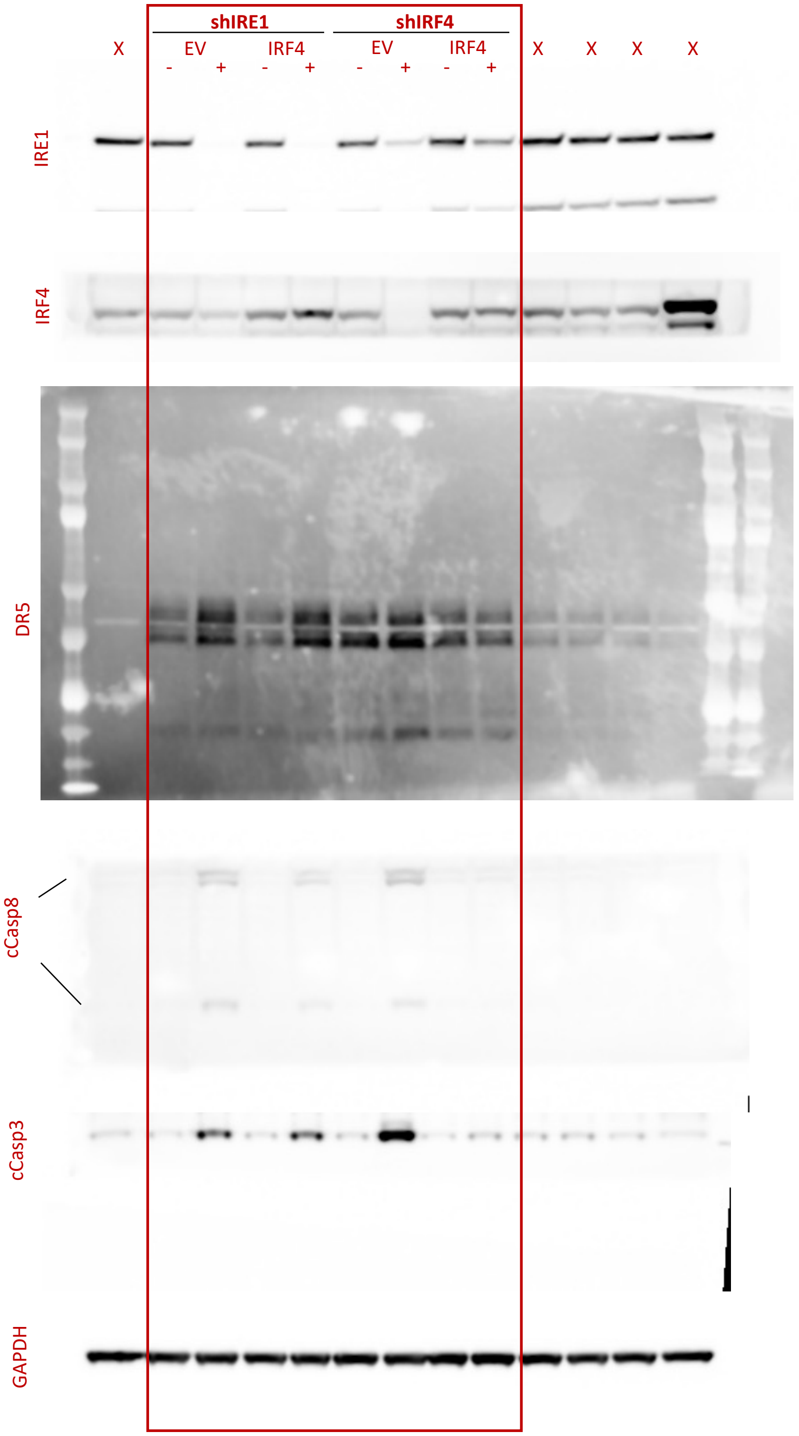
shIRE1
shIRF4
X
EV
IRF4
EV
IRF4
X
X
X
X
- + - + - + - +
IRE1
IRF4
DR5
cCasp8
cCasp3
GAPDH

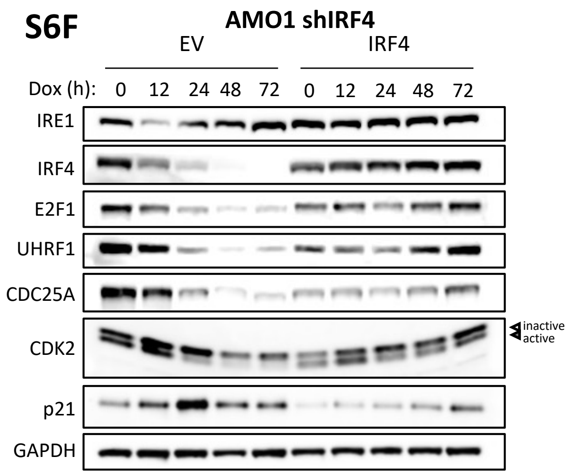
S6F
AMO1 shIRF4
EV
IRF4
Dox (h): 0 12 24 48 72 0 12 24 48 72
IRE1
IRF4
E2F1
UHRF1
CDC25A
CDK2
p21
GAPDH
inactive
active

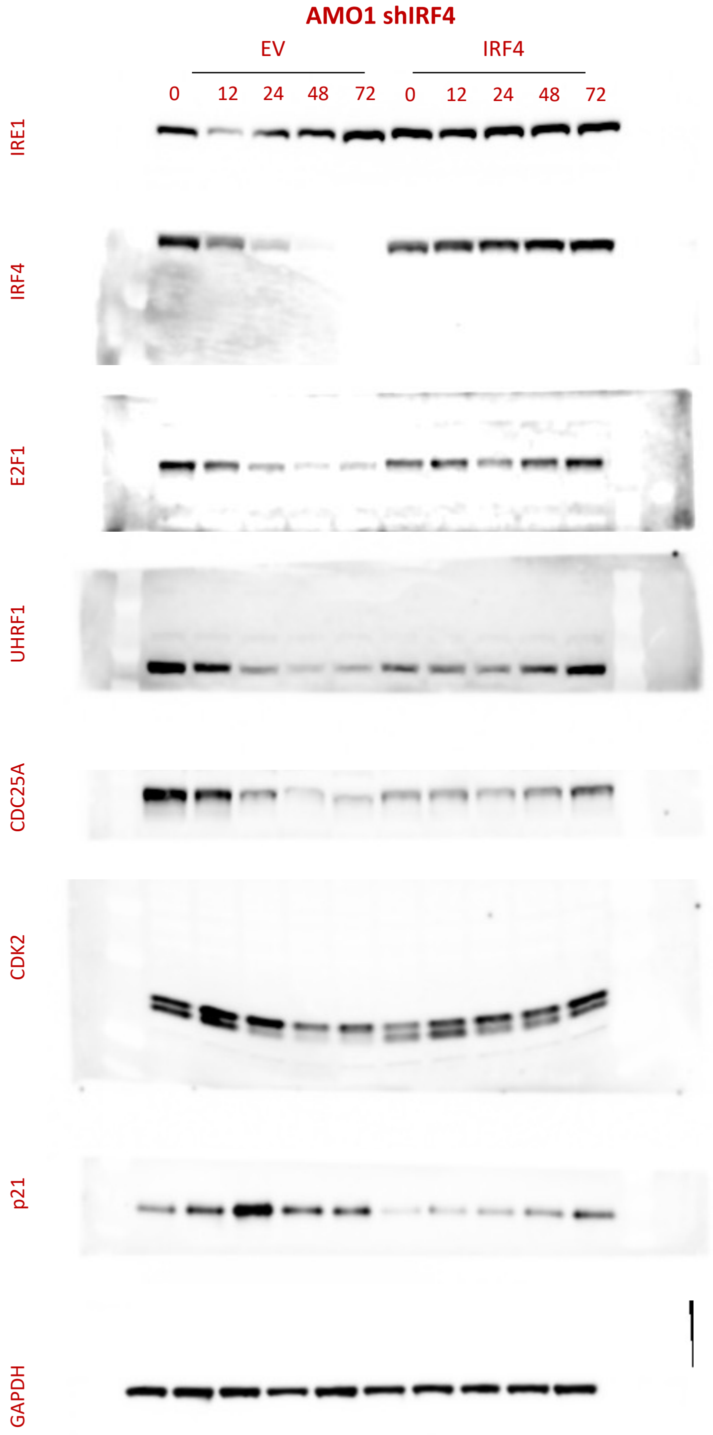
AMO1 shIRF4
EV
IRF4
0 12 24 48 72 0 12 24 48 72
IRE1
IRF4
E2F1
UHRF1
CDC25A
CDK2
p21
GAPDH

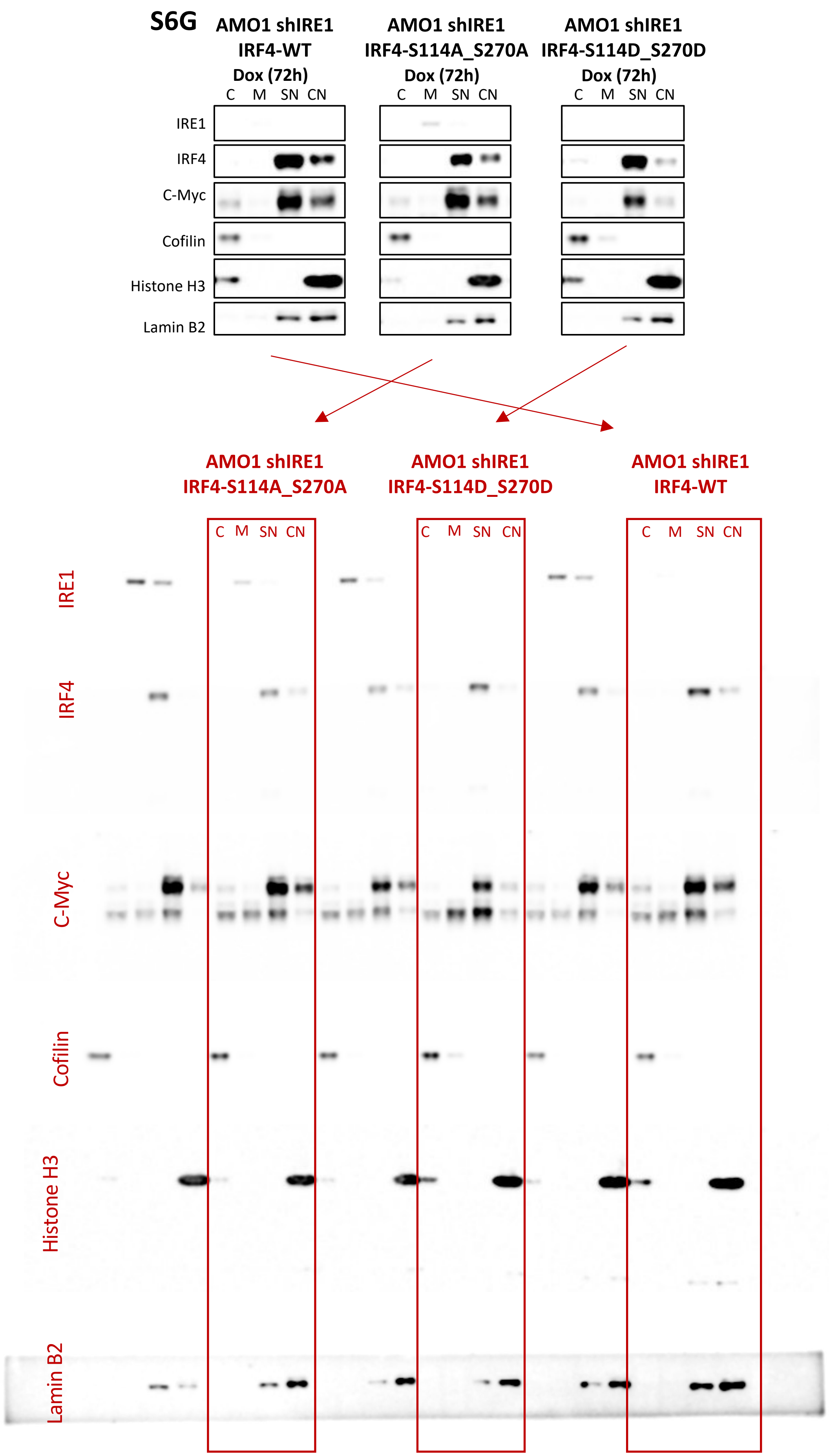
S6G
AMO1 shIRE1
IRF4-WT
Dox (72h)
C M SN CN
AMO1 shIRE1
IRF4-S114A_S270A
Dox (72h)
C M SN CN
AMO1 shIRE1
IRF4-S114D_S270D
Dox (72h)
C M SN CN
IRE1
IRF4
C-Myc
Cofilin
Histone H3
Lamin B2
AMO1 shIRE1
IRF4-S114A_S270A
AMO1 shIRE1
IRF4-S114D_S270D
AMO1 shIRE1
IRF4-WT
C M SN CN
C M SN CN
C M SN CN
IRE1
IRF4
C-Myc
Cofilin
Histone H3
Lamin B2
