# Supplementary material for: Interferon regulatory factor 4 mediates nonenzymatic IRE1 dependency in multiple myeloma cells
Source: PLoS Biol. 2025 Apr 11;23(4):e3003096. doi: 10.1371/journal.pbio.3003096 (PMC12052183; doi:10.1371/journal.pbio.3003096)
Supplement: Attachment — Submitted filename: PLOS Reviews Oikonomidi.docx [file pbio.3003096.s015.docx]

Dear Dr Ashkenazi,

Thank you for your patience while your manuscript entitled "IRF4 mediates nonenzymatic dependency on IRE1 in Multiple Myeloma cells" was peer-reviewed at PLOS Biology. It has now been evaluated by the PLOS Biology editors, an Academic Editor with relevant expertise, and by four independent reviewers.

The reviews are attached below. You will see that the reviewers find the conclusions novel and interesting, however they also think that more experiments are required to confirm the results and to obtain further mechanistic insights on how IRE1 works independently of its enzymatic functions and how critical is IRE1 for myeloma survival. Reviewer 1 wonders whether/how the relationship between IRE1 and IRF4 differs between MM cells that depend on IRE1 vs those that are not dependent on IRE1 activity (but dependent on the protein). Reviewers 2 and 3 think that additional experiments should be performed to confirm that IRE1 nonenzymatic activity is central to the proposed mechanism. These reviewers also suggest other experiments that include rescuing or exploring the role of miRNAs, confirming that IRE1 has a role in regulating nascent IRF4 mRNA and excluding the role of other transcription factors beyond their own transcriptional levels in the presence of IRE1 knockdown. In
addition, Reviewer 2 thinks the role of IRF4 phosphorylation in sustaining the generation of native IRF4 mRNA via the IRE1-driven chromatin binding of IRF4 should be clearly defined. Reviewer 4 asks for clarifications on the methods and other points that are important for understanding the conclusions, but also suggests an experiment to explore what happens to IRF4 when IRE1 is silenced in MM cell lines that are not dependent on IRE1 for their survival.

In light of the reviews, which you will find at the end of this email, we would like to invite you to revise the work to thoroughly address the reviewers' reports. Given the extent of revision needed, we cannot make a decision about publication until we have seen the revised manuscript and your response to the reviewers' comments. Your revised manuscript is likely to be sent for further evaluation by all or a subset of the reviewers.

We expect to receive your revised manuscript within 3 months. Please email us ([plosbiology@plos.org](mailto:plosbiology@plos.org)) if you have any questions or concerns, or would like to request an extension.

At this stage, your manuscript remains formally under active consideration at our journal; please notify us by email if you do not intend to submit a revision so that we may withdraw it.


**IMPORTANT - SUBMITTING YOUR REVISION**

Your revisions should address the specific points made by each reviewer. Please submit the following files along with your revised manuscript:

1. A 'Response to Reviewers' file - this should detail your responses to the editorial requests, present a point-by-point response to all of the reviewers' comments, and indicate the changes made to the manuscript.
*NOTE: In your point-by-point response to the reviewers, please provide the full context of each review. Do not selectively quote paragraphs or sentences to reply to. The entire set of reviewer comments should be present in full and each specific point should be responded to individually, point by point.
You should also cite any additional relevant literature that has been published since the original submission and mention any additional citations in your response.

2. In addition to a clean copy of the manuscript, please also upload a 'track-changes' version of your manuscript that specifies the edits made. This should be uploaded as a "Revised Article with Changes Highlighted" file type.

3. Resubmission Checklist
When you are ready to resubmit your revised manuscript, please refer to this resubmission checklist: <https://plos.io/Biology_Checklist>

To submit a revised version of your manuscript, please go to <https://www.editorialmanager.com/pbiology/> and log in as an Author. Click the link labelled 'Submissions Needing Revision' where you will find your submission record.

Please make sure to read the following important policies and guidelines while preparing your revision and fulfil the editorial requests:

a) *PLOS Data Policy*
Please note that as a condition of publication PLOS' data policy (<http://journals.plos.org/plosbiology/s/data-availability>) requires that you make available all data used to draw the conclusions arrived at in your manuscript. If you have not already done so, you must include any data used in your manuscript either in appropriate repositories, within the body of the manuscript, or as supporting information (N.B. this includes any numerical values that were used to generate graphs, histograms etc.). Please also indicate in each figure legend where the data can be found. For an example see here: <http://www.plosbiology.org/article/info%3Adoi%2F10.1371%2Fjournal.pbio.1001908#s5>

b) *Published Peer Review*
Please note while forming your response, if your article is accepted, you may have the opportunity to make the peer review history publicly available. The record will include editor decision letters (with reviews) and your responses to reviewer comments. If eligible, we will contact you to opt in or out. Please see here for more details:
<https://blogs.plos.org/plos/2019/05/plos-journals-now-open-for-published-peer-review/>

c) *Blot and Gel Data Policy*
Please provide the original, uncropped and minimally adjusted images supporting all blot and gel results reported in an article's figures or Supporting Information files. We will require these files before a manuscript can be accepted so please prepare them now, if you have not already uploaded them. Please carefully read our guidelines for how to prepare and upload this data: <https://journals.plos.org/plosbiology/s/figures#loc-blot-and-gel-reporting-requirements>

d) *Blurb*
Please also provide a blurb which (if accepted) will be included in our weekly and monthly Electronic Table of Contents, sent out to readers of PLOS Biology, and may be used to promote your article in social media. The blurb should be about 30-40 words long and is subject to editorial changes. It should, without exaggeration, entice people to read your manuscript. It should not be redundant with the title and should not contain acronyms or abbreviations. For examples, view our author guidelines: <https://journals.plos.org/plosbiology/s/revising-your-manuscript#loc-blurb>

e) *Protocols deposition*
To enhance the reproducibility of your results, we recommend that if applicable you deposit your laboratory protocols in [protocols.io](http://protocols.io/), where a protocol can be assigned its own identifier (DOI) such that it can be cited independently in the future. For instructions see: <https://journals.plos.org/plosbiology/s/submission-guidelines#loc-materials-and-methods>


Thank you again for your submission to our journal. We hope that our editorial process has been constructive thus far, and we welcome your feedback at any time. Please don't hesitate to contact us if you have any questions or comments.

Sincerely,

Ines
--
Ines Alvarez-Garcia, PhD
Senior Editor
PLOS Biology
[ialvarez-garcia@plos.org](mailto:ialvarez-garcia@plos.org)

------------------------------------

Reviewers' comments

Rev. 1:
This study is a follow-up to a parallel study (which I also reviewed) showing that a subset of multiple myeloma (MM) cells are dependent on IRE1 protein but not IRE1 enzymatic activity. The current work attempts to elucidate factors downstream of this non-enzymatic role of IRE1. Using a combination of transcriptomics and available data from DepMap, the authors identify IRF4 as one of the most consistently downregulated mRNAs in Ire-dependent MM cells. This is validated at the protein level, is shown to not be dependent on Xbp1s (and hence, is related to the non-enzymatic role of IRE1), and seems to mostly be at the level of transcription, with a partial contribution from increased mRNA decay. Further molecular analysis indicates that IRE1 somehow maintains IRF4 in a de-phosphorylated state, which allows it to both self-sustain its levels and act on downstream transcriptional targets that regulate cell cycle progression. Importantly, two lines of evidence are provided that
IRE1 is upstream of IRF4: (i) the set of genes affected almost completely overlaps in IRE1-lacking and IRF4-lacking cells; (ii) forced re-expression of IRF4 in IRE1-lacking MM cells rescues many of the key cell cycle consequences.

Overall, this study seems to me to be a major advance in two ways. First, it helps solidify the earlier conclusion that there is a non-enzymatic role for IRE1 in a subset of MM by now identifying a molecular player downstream of this unexplored pathway. Second, it highlights a key role for IRF4 in this subset of MM and provides a rich dataset of downstream consequences in this newly described IRE1-IRF4 axis. I am therefore supportive of publication. It is worth noting that signalling is not my area, and I cannot really evaluate the bioinformatics used in Fig. 1, but the robustness and quality of the data seem to be very high.

**We sincerely thank the reviewer for the careful and constructive review of both of our recent manuscripts. We appreciate the positive assessment of the work and are grateful for the insightful comments and recommendations.**

Only a couple of minor comments or suggestions:

1) The most important thing I was wondering throughout the manuscript was whether or how the relationship between IRE1 and IRF4 differs between MM cells that are dependent on IRE1 activity versus those that are not dependent on IRE1 activity (but nonetheless dependent on IRE1 protein). Can the authors comment on this? It could be useful in Fig. 1B to perhaps make the same plot for other MM cell lines where IRE1 activity is important. In short, I am wondering whether the authors think this alternative IRE1-IRF4 axis is only operating in the activity-independent MM cells, or is just more evident in these cells because the activity-dependent cells are confounded by other effects of IRE1.

**Thank you for directing our attention to this interesting question. To explore the regulation of IRF4 in a cell line that depends enzymatically on IRE1, we have now analyzed the well-characterized XBP1-dependent MM cell line KMS11 (**[**https://doi.org/10.1073/pnas.1906999116**](https://doi.org/10.1073/pnas.1906999116)**). First, as suggested, we now provide an equivalent graph to Fig 1B depicting the corrected gene effect plot for this cell line (Fig S1C). Second, we show that in KMS11 cells knockdown of either IRE1 or XBP1 leads to the downregulation of IRF4 (Fig S1D), which is consistent with enzymatic IRE1 regulation of IRF4 via XBP1s. We have noted this in the Results and Discussion. In addition, we show that in the IRE1-independent MM cell line, H929, as well as in an IRE1-independent T cell leukemia line, SR786, IRE1 silencing does not affect IRF4 levels (Fig. S1 E-H). Taken together with the other models we studied, this result suggests that the type of IRE1 dependency in a given cell line dictates the mode of IRF4 regulation.**

2) Some of the dot plots (e.g., Fig. 4E) and other graphs are very small and hard to read. Perhaps consider harmonizing the size of text and labels across all figures.

**Thank you for this observation. We have increased the font and symbol size wherever necessary to improve clarity.**

Rev. 2: Giovanni Tonon – note that this reviewer has signed his review.
In this manuscript, the Authors aim to define a mechanistic connection between the non-enzymatic activity of IRE-1 and IRF4, both central conduits in the hematolological cancer multiple myeloma (MM). The Authors argue that IRE1 is one of the main drivers of IRF4 activity, specifically on IRF4 regulation of the cell cycle. Much of the data on which the hypothesis of this manuscript rest is based on another manuscript, under consideration and not included. Nonetheless, from what it is possible to gather the results are interesting, and indeed provide useful mechanistic insights on the role of these two central players in MM, and their mutual interactions.

**We sincerely thank the reviewer for the careful and constructive review of our manuscript. We apologize for not including a link to the bioRxiv version of the preceding work. Please find it here** [**https://doi.org/10.1101/2023.11.22.567905**](https://doi.org/10.1101/2023.11.22.567905)

**A revision of that preceding manuscript is currently under review at PLOS Biology.**

Major points to be addressed:
1. the concept of nonenzymatic activity of IRE1 is central to this manuscript, yet it rests only on suppl fig. 1G-J, where an inhibitor has been used. To conclusively demonstrate this hypothesis, the Authors should reintroduce in cells knocked down for IRE1 either an shRNA-resistant wild type or inactive IRE1.

**We appreciate this helpful suggestion. The evidence provided in our preceding manuscript showed that in several MM cell lines Dox-induced IRE1 knockdown, but not the addition of an IRE1-specific RNase inhibitor (RI) and kinase inhibitor (KI), affected cell viability. For the reviewer’s convenience, we include this data herein (Fig A below, left panel). The proper inhibition of IRE1’s enzymatic activity by these compounds was validated by immunoblotting, which showed a loss in XBP1s and an increase in the RIDD target CD59 upon either IRE1 knockdown or small molecule inhibition (Fig A, right panel). In addition, in the revised version of that manuscript, we show that CRISPR/CAS9-based gene editing of the endogenous IRE1 locus to introduce a “kinase-dead” mutation (D688N), which prevents enzymatic IRE1 activity as confirmed by the loss of XBP1s and gain of CD59, permits essentially unimpeded proliferation (re-shown in Fig B below). Importantly, shRNA-based silencing of IRE1 D688N blocked proliferation, much like the knockdown of WT IRE1. Finally, also in the revised version of the preceding manuscript, we provide evidence that expression of shRNA-resistant WT and RNase-dead (K907A) IRE1 significantly and comparably rescued cell viability in the context of IRE1 knockdown (re-shown in Fig C below).**

**A**

**B**

**C**

2. the role of IRE1 in regulating not simply transcription, but nascent IRF4 mRNA is interesting, however only marginally addressed. It is unclear for example how the assessment of other transcription factors may affect this pattern. More thorough experiments, including rescue experiments, are required to both demonstrate a role for IRE1 in controlling nascent IRF4 mRNA, and to exclude the role of other transcription factors beyond their own transcriptional levels in the presence of IRE1 knock-down (as it was partially performed for MYC). For instance, the putative role of miRNAs, as suggested in the discussion, should be explored.

**We thank the reviewer for pointing out this important comment and the opportunity to improve the quality of the manuscript. Regarding other transcription factors, we have now assessed the activity of several factors known to regulate IRF4, namely, the negative regulator MITF, and the positive regulators Aiolos and Myc (Ikaros was not detectable), by analyzing chromatin binding through subcellular fractionation (Fig S2C). The results show that MITF and Aiolos do not display altered chromatin binding levels, whereas Myc binding to chromatin was decreased. Importantly, however, our Myc knockdown experiments already showed that Myc knockdown did not deplete IRF4 (Fig 2E; Fig S2D), indicating that Myc is not a key regulator of IRF4 regulation in this setting. In addition, we now provide further new evidence that Myc chromatin binding is similar upon rescue of IRE1 knockdown with WT or phospho-deficient IRF4 variants but diminished with a phospho-mimetic IRF4 mutant (Fig S6G-H), supporting the conclusion that IRF4 operates upstream of the observed changes in Myc.**

**Regarding rescue experiments addressing the role of IRE1 in regulating nascent IRF4 mRNA levels, we now provide additional data showing that phospho-mimetic IRF4 mutant (reflecting the gain of IRF4 phosphorylation caused by IRE1 depletion) fails to rescue nascent IRF4 mRNA transcription, whereas the corresponding phospho-deficient IRF4 mutant does provide a complete rescue (Fig 6J; Fig S6I).**

**Regarding miRNAs, we have examined miRNA17, for which reagents are available. The results (shown below) indicate that either IRE1 knockdown or XBP1s knockdown upregulates miRNA17, which excludes this miRNA from being a specific mediator of RIDD-dependent regulation of IRF4 by IRE1. Accordingly, we removed the statement about miRNA implication from the Discussion.**

**E**

**D**

**Panel D-E: *miR17* quantification during IRE1 or XBP1 silencing. AMO1 shIRE1 Cl.1 or shXBP1 Cl.1 cells were treated with or without 0.2 ug/mL Dox for 48 h before miRNA preparation. qPCR analysis was performed with miR103 (D) or miR16 (E) as internal controls.**

3. along these lines, it would be important to define the role of IRF4 phosphorylations in sustaining the generation of native IRF4 mRNA, through the IRE1-driven chromatin binding of IRF4.

**Thank you for this suggestion. We have added new data (Fig 2H; Fig S2E-F) showing that a phospho-mimetic IRF4 mutant (reflecting the gain of IRF4 phosphorylation caused by IRE1 depletion) fails to bind chromatin and reduces chromatin binding by endogenous IRF4 by ~50%. Furthermore, we performed a nuclear run-on experiment, which showed consistently with the chromatin binding data that the phospho-deficient mutant rescued (and almost doubled) nascent endogenous IRF4 mRNA expression, while the phospho-mimetic mutant failed to do so (Fig 6J). Please note that we can specifically detect endogenous IRF4 mRNA because our mutated IRF4 constructs are not recognized by the qPCR probes that are complementary to endogenous IRF4.**

4. It should be explained how the Ser114 as well as Ser270 on IRF4 have been selected.

**Thank you for suggesting this important clarification. We have now added this information in the results section. In brief, these two residues were identified in a phospho-proteomics mass-spectrometry experiment as the only IRF4 phosphorylations that were altered in a statistically significant manner upon IRE1 depletion but did not display significant change under pharmacological inhibition of enzymatic IRE1 activity (Fig 2G).**

5. it remains unclear why IRE1 and IRF4 knock-down triggers DNA damage. Could the Authors elaborate on this aspect?

**This is an interesting mechanistic question. Our RNA-seq results indicated downregulation of several DNA repair genes during IRE1 or IRF4 silencing. For example: subunits of the FANCM/CENPS complex that are responsible for DNA damage repair; several CDC and MCM genes, which support DNA integrity and genome stability; as well as multiple DNA polymerases and other enzymes (such as POLQ, DNA2) that specialize in DNA repair processes. In light of the reviewer’s question, we now include this data in Fig S4C-D and Fig S4F.**

6. how the Authors do intrepret the apparent lack of impact of knock-down of either IRE1 or IRF4 on the G2/M phase, in figure 3D? this finding seems at odds with the effect on G1 and S.

**Thank you for pointing this out. We believe this apparent inconsistency is an artifact of the analysis of non-synchronized cells. We have made it more explicit in the Results that we used pre-synchronized cells in order to better detect effects on G2/M. Indeed, this analysis shows that both the G1/S and G2/M populations are affected (Fig 3E).**

7. it is unclear the impact of IRF4 knock down on the ChIP-seq presented in figure 5A, as the profiles seem overlapping.

**We apologize for the poor quality of the initial data representation. We have replaced all the ChIP-seq panels with non-overlapping displays. Additionally, we have now reinforced our previous assumptions with an unbiased ChIP-seq differential binding analysis and *de novo* motif discovery (Fig 5A). The genomic regions identified by the differential binding analysis are highlighted with yellow shadowing on the updated genome browser profiles.**

Minor points:
- the dots in figure 3A are fairly difficult to read, please replace them with different colors.

**This has been improved although the overlap between several lines is inevitable.**

Rev. 3:
This is an interesting, but underdeveloped manuscript that reports that some multiple myeloma cell lines require the expression of the endoplasmic reticulum IRE1alpha kinase/RNase but neither of its enzymatic functions.

**We sincerely thank the reviewer for the assessment of our manuscript. We believe that our in-depth investigation amply meets objective standards for publication.**

The IRE1alpha/XBP1 pathway is known to be critical for normal plasma cell development. This pathway is often upregulated in myeloma and some studies (although not all) have shown that genetically or pharmacologically inhibiting this pathway blunts the growth of some myeloma cell lines.

Here, the authors make the interesting observation that a subset of myeloma cell lines fail to grow (and eventually die) when IRE1alpha is genetically silenced but not when XBP1 is genetically silenced. Hence, they appear to have a greater dependency on IRE1 than its canonical target XBP1. Moreover, they show that while genetic silencing of IRE1 affects the growth of these myeloma cell lines, pharmacologic inhibition of its kinase or RNase activities does not. Hence, this manuscript attempts to explore and mechanistically understand the requirement of these myeloma cell lines for the nonenzymatic function of IRE1.

By examining data from the DepMap.Org Portal and their own transcriptomics results, they focused their examination on Interferon Regulatory Factor 4 (IRF4) as a possible downstream target of IRE1. IRF4 has been long known to be highly expressed in B cells and plasma cells and plays essential roles in controlling B cell to plasma cell differentiation and immunoglobulin class switching. IRF4 overexpression is often found in myeloma cells and required for their survival. This makes it an interesting topic of study here, but also a challenging one because it plays a critical role in myeloma cell survival and manipulating it likely has effects independent of IRE1.

They author show here that shRNA silencing of IRE1 but not XBP1 significantly downregulated the IRF4 mRNA and protein levels in AMO1, KMS27, and L363 cells. They fail to test what happens to IRF4 in myeloma cell lines that are dependent on IRE1 enzymatic activity and XBP1 expression, which should be done for comparison.

**Thank you for this suggestion. We have now performed additional experiments with the KMS11 cell line, previously documented to depend enzymatically on IRE1 and on XBP1s (**[**https://doi.org/10.1073/pnas.1906999116**](https://doi.org/10.1073/pnas.1906999116)**). The results show that in KMS11 cells knockdown of either IRE1 or XBP1s downregulates IRF4 (Fig S1D), indicating that XBP1s is the regulatory conduit between IRE1 and IRF4 in a context that requires enzymatic IRE1 activity. We also included new data indicating that IRE1 does not affect IRF4 levels in IRE1-independent H929 and SR786 cells (Fig. S1 E-H).**

Nascent-mRNA analysis showed that IRE1 silencing significantly reduced de novo IRF4 transcription, but they were not able to connect this with any known IRF4-regulating transcription factors. Since IRF4 is an auto-stimulatory transcription factor, they explored the possibility that IRE1 regulates IRF4's activity itself. They performed subcellular fractionation and found that IRE1 silencing markedly decreased the relative amount of IRF4 bound to chromatin, suggesting a functional disruption of IRF4 activity. However, this is difficult experiment to quantify in this way and ChIP would be a better method.

**We understand the reviewer’s concern. However, we think that our chromatin binding analysis is in fact a more accurate approach than ChIP because it enables a more direct, side-by-side quantification of the nuclear IRF4 protein that is either bound or not bound to chromatin for each specific experimental treatment. The approach we used helps discern the relative changes in chromatin binding while accounting for differences in the total level of the transcription factor examined. In contrast, ChIP would reflect the absolute chromatin-bound protein under different experimental treatments. The latter strategy would be impacted by changes in protein levels, nuclear localization, as well as activity, making it less optimal for gauging IRF4’s specific activity. Please note also that our finding is now further corroborated by the new results with IRF4 phosphorylation mutants – particularly the phospho-mimetic mutant, which phenocopied chromatin binding by IRF4 under IRE1 knockdown (Fig 2H; Fig S2E-F).**

The authors then present evidence that IRE1 silencing significantly increased IRF4 phosphorylation on Ser114 as well as Ser270, while pharmacologic IRE1 inhibition did not induce a significant increase. However, the quantification methods used are not well explained and make it difficult to assess confidence in the results. I am not sure if any phospho antibodies exists against these sites, but the authors do not assess them by immunoblotting.

**We apologize for not including sufficient information. We performed the phospho-proteomics analysis using a well-established approach, and the detection of phosphorylation sites via mass spectrometry is considered to be highly sensitive and reliable (**[**https://doi.org/10.1016/j.jbc.2022.102729**](https://doi.org/10.1016/j.jbc.2022.102729) **and** [**https://doi.org/10.1016/j.jbc.2022.102729**](https://doi.org/10.1016/j.jbc.2022.102729)**)**. **Additionally, we performed a global proteomic analysis by mass spectrometry to normalize phosphorylation levels against total protein levels. We have now included this information for clarification in the figure legend. More information can be found in the materials and methods section.**

**We agree that IRF4 phospho antibodies would be useful; unfortunately, they are not available as yet. As an orthogonal validation for the phospho-proteomics results, we have performed phostag-gel electrophoresis, where we can visualize changes in the IRF4 phosphorylation status during IRE1 knockdown (panel F, below), which confirmed the appearance of new, λ-phosphatase susceptible, phospho-IRF4 species. We hope that the publication of our results would facilitate the future generation of such reagents by entities that specialize in this type of endeavor.**

**Panel F: Detection of IRF4 phospho-species by Photag gel electroporation. AMO1 shIRE1 Cl.1 cells were treated with 0.2 ug/mL Dox for 0, 24 or 48 h. The resulting lysates were then treated with or without λ-phosphatase. The lysates were then normalized for IRF4 protein content (instead of total protein content) in order to boost detection efficiency and loaded on a phostag-containing gel (Wako). Samples treated with λ-phosphatase serve as negative phosphorylation controls.**

A functional consequence of IRF4 phosphorylation on Ser114 or Ser270 does not seem to have been previously reported in the literature (although p38a MAPK has been reported capable of phosphorylating Ser270--PMID: 36443297). Therefore, in an attempt to examine the functional importance of these phosphorylation sites, the authors generated an IRF4 mutant in which these serine residues were replaced with alanine (S114A_S270A). Upon ectopic expression in AMO1 cells, this mutant displayed a 4-fold increase in its chromatin-binding capacity as compared to WT IRF4, indicating that phosphorylation on these sites inhibits IRF4 activity. However, as the ectopically expressed proteins are run on separate gels (**Fig S2D**), we are unable to assess whether their expression is comparable. Moreover, as they did not carry out transcriptional studies with these mutants we have no direct evidence on how these mutations affect their transcriptional activity. Moreover, they did not compare how
IRF4 wt vs non-phosphorylatable mutants are able to rescue IRE1 depletion in myeloma cells. If their model is correct, IRE1 depletion should have negligible effects on cells expressing the IRF4 non-phosphorylatable mutant--an important experiment that was not done.

**Thank you for this important set of comments. We have now added the required control data as requested (Fig S2E; Fig 6K), showing that the expression of WT vs phospho-mutant IRF4 species is equivalent in both AMO1 as well as AMO1 shIRE1 cells.**

**We have also performed additional experiments with shRNA-resistant phospho-deficient as well as phospho-mimetic mutants of IRF4. The results show that in the context of IRE1 knockdown the phospho-mimetic mutant (reflecting the gain of IRF4 phosphorylation caused by IRE1 depletion) fails to rescue cell proliferation and cell cycle-arrest of IRE1 deficient cells (Fig 6H-I). Importantly, phospho-mimetic IRF4 expression in IRE1-deficient cells had no effect on the levels of E2F1, Cdc25A and UHRF1, which we identified as important nodes downstream to IRF4, both at the RNA and protein level (Fig 6J-K; Fig S6I). On the contrary, phospho-deficient IRF4 does provide a complete rescue of all these effects of the IRE1 depletion (Fig 6H-K; Fig S6G-I).**

The authors then carry out a series of studies comparing IRF4 knockdown to IRE1 knockdown, find that both impair myeloma cell growth, and try to build a case that they are in linear pathway. Unfortunately, these experiments are not convincing given that loss of two parallel but important growth pathways can give similar effects on cell growth. These experiments are not particularly helpful or enlightening.

**Thank you for raising this point. Although there is a formal possibility that IRF4 rescues proliferation in the absence of IRE1 by acting in parallel to IRE1 rather than downstream, our conclusion that IRE1 and IRF4 act in a “linear” pathway is based on the integration of several lines of evidence. (1) IRE1 knockdown downregulates IRF4 mRNA and protein (Fig 1A and C-F; Fig S1A-D); (2) IRE1 knockdown inhibits IRF4 chromatin binding in a manner that depends on IRF4 phosphorylation sites controlled by IRE1 (Fig 2F-H; Fig S6G,H); (3) the cell-cycle and related transcriptional phenotypes of IRE1 and IRF4 depletion closely resemble one another (Fig 3; Fig 4); (4) successful rescue of IRE1 silencing by IRF4 re-expression depends on the specific IRF4 phosphorylation sites that IRE1 controls (Fig 6H-K; Fig S6 H-J); (5) ectopic IRF4 overexpression did not provide an added proliferative benefit beyond endogenous IRF4 (Fig 6B; Fig 6E). Please also consider the following comment made by Reviewer #1: “Importantly, two lines of evidence are provided that IRE1 is upstream of IRF4: (i) the set of genes affected almost completely overlaps in IRE1-lacking and IRF4-lacking cells; (ii) forced re-expression of IRF4 in IRE1-lacking MM cells rescues many of the key cell cycle consequences”.**

In Figure 6, the authors finally carry out their first IRF4 repletion studies to test if this rescues cells deficient in IRE1. IRF4 repletion during IRE1 silencing was sufficient to rescue proliferation of AMO1 cells (Fig 6A-B). Although IRF4 re-expression effectively restored growth for up to 72 h, a modest growth decline of up to 15% occurred beyond this time point. As mentioned, these are difficult experiments to interpret because it is possible that IRF4 expression may very well promote cell growth (as it is known to do), but does not mean that it is downstream of IRE1. Moreover, this is the experiment where the IRF4 non-phosphorylatable mutant should also be tested. Furthermore, IRF4 repletion only partially prevented apoptosis induction during IRE1 silencing, as measured by DNA fragmentation (Fig 6C) or caspase-3/7 activation, against suggesting that it is not sufficient.

**While IRF4 can promote proliferation, there is published evidence that it can act also as a tumor suppressor (**[**https://doi.org/10.1038/s41388-020-01438-1**](https://doi.org/10.1038/s41388-020-01438-1) **and** [**https://doi.org/10.1200/jco.2014.32.15_suppl.620**](https://doi.org/10.1200/jco.2014.32.15_suppl.620)**). Also, as noted above, ectopic IRF4 overexpression did not provide an added proliferative benefit beyond endogenous IRF4 (Fig 6B; Fig 6E). Together with the other lines of evidence listed in the response to the previous comment, the results strongly support our conclusion that IRF4 operates downstream rather than in parallel to IRE1.**

**Regarding the IRF4 mutant, as noted above, we have included new results for both non-phosphorylatable (phospho-deficient) and phospho-mimetic IRF4 mutants (Fig 6H-K; Fig S6G-I).**

**As for the inability of IRF4 repletion to prevent apoptosis, we now provide additional data indicating that IRE1 knockdown upregulates the death receptor DR5 (Fig S6D), previously linked as a RIDD target to apoptosis regulation under ER stress (see Results for references). As expected, IRF4 repletion did not prevent DR5 upregulation, consistent with IRF4’s inability to recover IRE1 enzymatic activity. This new observation may explain the lack of complete apoptosis prevention by IRF4 repletion.**

In the end, the reader is left with more questions than answers. While the data suggest that a subset of myeloma cell lines require some nonenzymatic function of IRE1, the effects on IRF4 loss are largely phenomenological at this point. it is unclear if they are in a linear pathway. It is unclear if/how IRE1 regulates IRF4 mRNA levels and/or phosphorylation.

As such, this manuscript does not provide a substantial enough advance to justify publication in PLoS Biology.

**Our additional experiments strengthen the conclusion that IRE1 loss affects specific IRF4 phosphorylation sites in a manner that impacts IRF4’s chromatin binding activity, self-regulation, transcriptional activity, and cell-cycle control. Reporting these findings to the scientific community will open the door for further mechanistic investigation of how IRE1 nonenzymatically controls IRF4.**

Rev. 4:
The paper by Oikonomidi and colleagues examines the basis for a non-enzymatic dependency on IRE1 in a subset of MM cell lines and suggests that the dependency is mediated by the transcription factor IRF4. Here the authors begin by identifying a set of genes that are essential for the survival of IRE1-dependent MM cell lines and filterred this list for those that are transcriptionally dependent on IRE1 for expression. Through this approach, the authors identified IRF4 (among others) as a key target that was downregulated upon IRE1 silencing. From here, the authors then perform a large series of experiments to investigate the basis for the association between IRE1 and IRF4. The most novel of these is the finding that IRE1 silencing leads to the phosphorylation of IRF4, which disrupts the ability of IRF4 to engage DNA and drive transcription. The authors also show that IRF4 overexpression can rescue the anti-proliferative effect that is seen with the loss of IRE1.

Overall, the manuscript is well written, the experiments are well executed and extensive, and the findings, albeit in a select group of cell lines, will be of interest to the myeloma research community. Additionally, in the discussion, the authors acknowledge that further work is required to decipher just how IRE1 regulates IRF4 phosphorylation. There are a few questions however that this reviewer would like to see addressed. The most notable of these is the disconnect between how IRE1 is presumably only required for the survival for a small subset of MM cell lines, yet IRF4 is required for all.

**We sincerely thank the reviewer for the careful and constructive review of our manuscript. We appreciate the positive assessment of the work and are grateful for the insightful comments and recommendations.**

Questions.
1. The authors should state how many MM cell lines (proportion of the ones they tested) they found to be IRE1 dependent. This is important as the Depmap data that they use extensively throughout the manuscript would argue that IRE1 loss has little effect on MM survival.

**We have added this information in the results section (Fig S1H). Briefly, out of 7 MM cell lines tested, AMO1, KMS27, L363, JJN3, KMS11 and OPM2 were IRE1 sensitive while only H929 was resistant.**

2. Additionally, the authors should indicate how many of the IRE1-dependent MM cell lines were independent of IRE1's enzymatic activity. This subset is likely even smaller, and this detail would add clarity to their findings.

**As now clarified in a new schematic (Fig S1I), 4/6 IRE1-dependent MM cell lines require IRE1 nonenzymatically.**

3. In Figure 1, the authors exclude common essential genes from their analysis. They should provide a rationale for this decision. In Figure 2D, IRE1 silencing leads to the downregulation of the common essential gene MYC. Could MYC downregulation be driving the loss of cell viability independently of IRF4?

**Thank you for bringing this to our attention. We apologize for any confusion: we did not filter out the “common essential genes” provided by Depmap. We have now modified the Methods section and the figure legends describing the corrected gene effect score calculation to facilitate clarity. In brief, we first calculated the average Chronos score of each gene across all the cell lines analyzed by Depmap (n=1101). This correction was meant to blank the scores of pan-essential genes and to accentuate the MM-specific dependencies. Subsequently, in order to find nonenzymatic-IRE1 codependencies, we filtered this list further for essentiality (corrected score ≤-0.25) in all nonenzymatic-IRE1 dependent cell lines (Table S1, 131 genes). Importantly, this list of 131 genes still contains Myc, albeit not with a high corrected score (Table S1). This is in agreement with the “preferentially essential gene scores” (z-scores) provided by Depmap (Table below). Hence, IRF4 is deemed of greater importance than Myc in these cell lines.**

| **Corrected Gene Effect of IRE1-codependencies (AMO1)** | | | **Preferential Essentiality (AMO1)** | | |
| --- | --- | --- | --- | --- | --- |
| **Rank** | **Gene** | **Corrected Score** | **Rank** | **Gene** | **z score** |
| 1 | IRF4 (3662) | -1.7768802 | 26 | IRF4 (3662) | -4.4944493 |
| 57 | MYC (4609) | -0.785867 | 2687 | MYC (4609) | -1.09835 |

**Despite this lower corrected (i.e., calculated) gene essentiality of MYC in MM, we agree that it should not be overlooked. Indeed, the Chronos effect of MYC is -2.48, which is lower than the corresponding value of -1.86 for IRF4. Accordingly, we further assessed the effect of Myc loss (Fig S3M). Our data does show that depletion of Myc decreases proliferation; however, as expected from the above, this effect is not as strong as that of IRE1 or IRF4 knockdown. Additionally, Myc depletion had only a minimal cell-cycle arrest. Thus, although Myc likely contributes to growth downstream of IRE1, it does so to a lesser degree than IRF4. Notably, IRE1 silencing negatively impacted Myc chromatin binding (Fig S2C), possibly affecting its transcriptional activity as well as levels. Importantly, IRF4 silencing itself led to Myc downregulation (Fig 4A, 4D, 4E), while re-expression of phospho-deficient but not phospho-mimetic IRF4 rescued Myc chromatin binding (Fig S6G-H). Altogether, these results indicate that Myc is primarily a target of IRF4 in the context of IRE1 silencing.**

4. The authors also identified the key MM survival gene PRDM1 as an IRE1-dependent gene? The authors should provide a rationale as to why they focused on IRF4 and not PRDM1. It may be that they investigated PRDM1, and it led to a dead end.

**We did not pursue PRDM1 for the reason outlined below, albeit second on the list of IRE1-regulated essential genes in MM lines with nonenzymatic IRE1 dependency (Fig 1B). First, IRF4 displays the highest gene effect in these cell lines (Fig 1B). Furthermore, IRF4 is widely recognized as a de-repressor of PRDM1 (please see references 16, 17, 20 and 22 of the manuscript). The latter is corroborated by our RNAseq, ChIPseq and rescue data (Fig S4A; Fig S5B; Fig S6I). Notably, both the timeframe and extent of PRDM1 depletion upon IRE1 loss (Fig 1A) support the possibility that IRF4 downregulation mediates PRDM1 loss in the context of IRE1-deficiency.**

5. The authors show that IRE1 silencing leads to the loss of both IRF4 transcript and protein in IRE1-dependent MM cell lines. What happens to IRF4 when IRE1 is silenced in MM cell lines that are NOT dependent on IRE1 for their survival. Including additional data here would significantly strengthen the paper and allow the authors to make the case that for this subset of MM cell lines, IRE1 is somehow uniquely important for IRF4 maintenance.

**Thank you for your valuable thoughts on how to strengthen the impact of this manuscript. We have now included data from the IRE1-independent MM cell line, H929, as well as a T-cell lymphoma, SR786, that expresses high levels of IRF4 and lower levels of IRE1 (Fig S1E-H). Our data shows that IRF4 is unaffected by IRE1 silencing in IRE1-independent cell lines, supporting the idea that IRE1 is uniquely important for IRF4 maintenance in the subset of IRE1-dependent cell lines.**

6. Finally, in the discussion, the authors should address the apparent discrepancy between IRE1's requirement in a subset of MM cell lines and IRF4's essentiality across all MM cell lines.

**We thank the reviewer for requesting this important clarification. IRF4 displays wider essentiality than IRE1. However, in a subset of MM, IRE1 plays a key role in supporting IRF4 expression and activity. In the other subset, some other factor(s) control IRF4. (For example, in H929, an IRE1-independent cell line, Myc plays a central role in regulating IRF4.) The former subset can be further divided into cell lines in which IRE1 controls IRF4 enzymatically (i.e., via XBP1s; e.g., KMS11) or nonenzymatically (i.e., via post-translational modification of specific phosphorylation sites; e.g., AMO1). We now include a schematic model that further clarifies this relationship (Fig S7).**
